# The Content of Our Cooperation, Not the Color of Our Skin: An Alliance Detection System Regulates Categorization by Coalition and Race, but Not Sex

**DOI:** 10.1371/journal.pone.0088534

**Published:** 2014-02-10

**Authors:** David Pietraszewski, Leda Cosmides, John Tooby

**Affiliations:** 1 Center for Evolutionary Psychology, University of California Santa Barbara, Santa Barbara, California, United States of America; 2 Department of Psychological & Brain Sciences, University of California Santa Barbara, Santa Barbara, California, United States of America; 3 Department of Anthropology, University of California Santa Barbara, Santa Barbara, California, United States of America; Harvard University, United States of America

## Abstract

Humans in all societies form and participate in cooperative alliances. To successfully navigate an alliance-laced world, the human mind needs to detect new coalitions and alliances as they emerge, and predict which of many potential alliance categories are currently organizing an interaction. We propose that evolution has equipped the mind with cognitive machinery that is specialized for performing these functions: an *alliance detection system.* In this view, racial categories do not exist because skin color is perceptually salient; they are constructed and regulated by the alliance system in environments where race predicts social alliances and divisions. Early tests using adversarial alliances showed that the mind spontaneously detects which individuals are cooperating against a common enemy, implicitly assigning people to rival alliance categories based on patterns of cooperation and competition. But is social antagonism necessary to trigger the categorization of people by alliance—that is, do we cognitively link A and B into an alliance category only because they are jointly in conflict with C and D? We report new studies demonstrating that peaceful cooperation can trigger the detection of new coalitional alliances and make race fade in relevance. Alliances did not need to be marked by team colors or other perceptually salient cues. When race did not predict the ongoing alliance structure, behavioral cues about cooperative activities up-regulated categorization by coalition and down-regulated categorization by race, sometimes eliminating it. Alliance cues that sensitively regulated categorization by coalition and race had no effect on categorization by sex, eliminating many alternative explanations for the results. The results support the hypothesis that categorizing people by their race is a reversible product of a cognitive system specialized for detecting alliance categories and regulating their use. Common enemies are not necessary to erase important social boundaries; peaceful cooperation can have the same effect.

## Introduction

A common threat, history shows, can create new cooperative alliances. The goal of defeating a common enemy is so powerful an organizing force that it can bring together individuals from groups, factions, and coalitions that are usually estranged or even in conflict. As Aristotle observed, “a common danger unites even the bitterest enemies.”

What psychological systems underwrite this unifying process? Converging evidence from several fields—evolutionary psychology, cognitive development, social psychology, evolutionary game theory, and behavioral economics—suggest that coalitional cooperation is orchestrated by a suite of cognitive specializations that evolved for this function, which organize how we perceive situations and frame issues involving cooperative alliances [1]–[10]. Here we report experiments testing the proposal that this suite of adaptations includes *an alliance detection system*: a neurocognitive system that is specialized for tracking alliances and retrieving those most likely to be useful for understanding situations as they unfold [1], [2], [11], [12].

By hypothesis, the alliance detection system performs a specialized form of social categorization. It has encoding functions that selectively attend to patterns of coordinated action, cooperation, and competition, such as when X works at Y's side, supports Y's side in a dispute, sacrifices to help Y, or stands shoulder to shoulder with Y against an enemy. Based on these patterns, the system infers who is allied with whom with respect to specific issues, implicitly assigning individuals to coalitions or alliance categories. This category information is stored for later use, along with any cues that predict alliances—manner of dress, gait, speech, family resemblance, and so on—so that potential allies can be identified in advance of organizing a group project or recruiting support in response to a threat.

Because each individual belongs to more than one alliance category, the alliance system requires retrieval functions that deliver the right alliance categories to the right decision rules at the right time. This requires dynamic Bayesian up-dating. The retrieval system should use past experiences to compute the prior probability that specific alliance categories will be relevant in a well-defined context (or even across situations). These priors will regulate which alliance categories the system retrieves when no alternative information is available. But as an event unfolds, these default values should be updated dynamically, on the basis of cues indicating which alliances are most relevant to the individuals involved. The alliance system should use these revised probabilities to up-regulate the retrieval of those coalitions or alliances that are most likely to be organizing people's behavior as the situation develops, and down-regulate the retrieval of less useful alliance categories.

The social consequences of a system with this design are interesting. It will create alliance categories that reflect long-standing social divisions, like those based on race or social class, and retrieve them easily. In this view, racial categories, such as *black* and *white*, arise in the cognitive system because they function as alliance categories. They are retrieved strongly across situations because the system has computed a high prior probability of their predicting patterns of alliance. But—contrary to prevailing views—it should be easy to down-regulate the retrieval of these categories by manipulating alliance-relevant variables. For example, when the goal of defeating a common enemy makes a new, more inclusive alliance category highly relevant, social categories such as *black* and *white* may become cognitively irrelevant—at least temporarily.

Recent events in the United States are suggestive: One month after Al-Qaeda terrorists attacked the World Trade Center, a *New York Times* headline read “Sept 11 attack narrows the racial divide”. The story reported “a new spirit of cooperation” between black and white New Yorkers [13]. Echoing Aristotle, a group of black and Latino boys who used to view the neighborhood police as “enemies” said that they and the (mostly white) officers no longer view each other with suspicion. The attack changed, at least temporarily, how the boys categorized themselves and others: “The boys see themselves transformed. ‘I just thought of myself as black,’ [one] said. ‘But now I feel like I’m an American, more than ever.’” Cognitively speaking, they had redrawn the lines defining *us* and *them*.

### War and peace

But does it take an attack of this magnitude to elicit the formation of new alliance categories and make race fade in relevance? More importantly, can peaceful cooperation achieve the same effects? This is the central question we address herein.

A standard method, the “Who said what?” paradigm [14], allows researchers to unobtrusively measure social categorization under controlled laboratory conditions. Our studies use this method to test the alliance detection hypothesis, because it can sensitively measure shifts in how people categorize their social world. We build on early studies by Kurzban, Tooby, and Cosmides [1], which tested situations in which race did not predict coalition membership, but patterns of cooperation and competition did. Those studies showed that coalitional conflicts—even minor ones—can elicit the formation of new alliance categories. When the new alliance categories were made visually-distinct (by introducing a correlated difference in shirt color—a shared appearance cue), categorization by coalition increased and categorization by race decreased. Only four minutes of exposure was necessary to make racial categories temporarily irrelevant.

The experiments we report challenge the assumption that antagonism between rival coalitions—common enemies—is necessary to activate alliance detection and decrease racial categorization. If an alliance detection system exists, then friendly interactions between two coalitions, in which race does not predict alliances, should melt racial boundaries just as effectively as coalitional conflict. Instead of two warring coalitions, our studies present subjects with two charity groups—*Habitat for Humanity* and *Partners in Health—*whose members are having an amiable conversation, sharing information about how each group cooperates to help people.

Using this scenario of peaceful cooperation, we test major features of the alliance detection system, including its scope, retrieval functions, and independence from other cognitive systems that regulate social categorization.

### Measuring social categorization: The “Who said what?” paradigm

The “Who said what?” (WSW) paradigm unobtrusively reveals whether observers of a social interaction have implicitly categorized the individuals involved, and how strongly they did so. The social interaction can be a conflict between two rival coalitions, a housewarming party, a chat between the members of two charity groups, or anything other scenario of interest to the experimenter. Before it starts, study participants are asked to watch and form impressions of the people involved.

As the scenario unfolds, these participants see eight speakers make a series of comments. The speakers vary in ways of interest to the experimenter; the differences may be apparent from their photos (e.g., sex, race, age) or from what they say (e.g., interests, attitudes, affiliations). When the scenario is over, participants are given a surprise memory task: they are asked to recall who said what. A display with the eight photos appears, and comments from the scenario are presented, one at a time, in random order. As each comment appears, the participant indicates which individual said it (by choosing a photo). There are usually at least 24 comments to attribute, so the participants make many mistakes.

Their mistakes reveal how they categorized the speakers. When a cognitive system in the observer has been assigning individuals to categories, the observer will be more likely to confuse members of the same category (e.g., two women) than members of contrasting categories (e.g., a woman and a man). This bias will be apparent when participants attribute comments to the wrong speaker.

Consider, for example, participants who have not encoded the sex of the speakers at all. Their recall errors will be random with respect to sex, so there will be no significant difference in the rate at which they confuse same-sex and opposite-sex speakers; the same will be true of participants who encoded the sex of the speakers, but did not use gender as a retrieval cue at recall. But when participants make significantly more within-sex errors than between-sex errors, one can infer that they not only encoded the sex of the speakers, but also used gender as a retrieval cue when asked to recall who said what. The more within-sex errors are made relative to between-sex errors, the more strongly participants categorized the targets by their sex. A significant difference in the two error rates reveals whether speakers were categorized by their sex at all. The effect size associated with this difference provides a quantitative measure of how strongly they were categorized. Both indicators—significance and effect size—are important in testing predictions of the alliance detection hypothesis.

### Visually-distinct social categories

The WSW method was first used to investigate visually-distinct social categories—ones that can be discerned from photos, such as sex, age, race, and manner of dress. Years of research using this instrument have established that people spontaneously categorize newly encountered individuals by their sex, age, and race [14]–[21]. Effect sizes for all three categories are usually large, even when they are not relevant to the scenario tested. Sex plays a role in our studies as a contrast category; by hypothesis, racial categories are primarily alliance categories, regulated by the alliance system, but gender categories are not (see below).

Mentally classifying people as *black* or *white* need not activate racial stereotypes or discriminatory behavior, but it is a precondition for both. For this reason, there was a concerted effort to find social contexts that would reduce racial categorization, most of which failed [14], [16], [19], [22]. Racial categorization remained high despite clever manipulations of social context, topic of conversation, attention, instructions, and cognitive load—factors that are known to decrease racial stereotyping [23]–[28]. The retrieval of racial categories is easy to trigger and difficult to suppress [22].

This discovery informed our decision to use racial categories to test properties of the alliance system: Down-regulating their retrieval is a tough test for any theory to pass. The experiments that failed had one notable feature in common—they did not manipulate cues that should regulate the retrieval of alliance categories.

### Coalitional conflict or visually-distinct categories?

For the alliance detection system to revise a high prior probability that race is relevant for predicting alliances, the social context should provide cues that alliance categories *other than* race are organizing people's behavior. That is what the coalitional conflict studies conducted by Kurzban et al. [1] did.

The speakers were eight men, four black and four white, who belong to two rival coalitions (basketball teams that fought last season). These two social categories were crossed, such that each coalition had two black and two white members. Study participants knew they would see a conversation between members of two teams, but their racial composition was never mentioned.

In the first study, race was the only visually-distinct social category because all the men were wearing identical gray shirts (Exp 1; *gray condition*). Participants watched a heated argument between members of the two coalitions. As the men traded insults and accusations, their patterns of agreement and disagreement provided a basis for inferring who was allied with whom. In response to this unfolding pattern, participants spontaneously categorized the men by their coalitional alliances, even though there were no visual distinctions marking team membership (effect size: *r* = .31). They also categorized the men by race (*r* = .67).

To make the coalitional categories as visually-distinct as racial categories are, the experimenters ran a second condition that differed in only one respect: men from one coalition wore gray shirts and men from the other wore yellow ones (Exp 2; *color condition*). Adding a visual distinction that tracks patterns of cooperation and competition should make it easier for the alliance system to detect that race is not organizing behavior in this particular conflict, and that other cooperative alliances are. As predicted, making the alliance categories visually-distinct increased coalitional categorization (to *r* = .79) and decreased racial categorization (to *r* = .49). In a replication using different men and shirt colors as targets (Exps 5, 6), the same manipulation produced a drop in racial categorization from *r* = .57 to *r* = .15. In this last case, race was “erased”—there was no statistically detectable categorization by race.

### Eliminating alternative explanations

Was this decline produced by an alliance system, or were other factors at work? Finding that coalitional variables regulate race, but not gender, could rule out many alternative hypotheses. When coalition membership was crossed with sex (Exps 3, 4), the results for coalitional categorization were virtually identical to those found when coalition was crossed with race (*gray*: *r* = .35; *color*: *r* = .81). But gender categorization never decreased to the levels found for racial categorization (*gray*: *r* = .91; *color*: *r* = .84).

These results demonstrate that attention and working memory have enough capacity to support the strong retrieval of two crossed categories. Sex and coalition were both retrieved at high levels in the color condition (*r*s = .84, .81), so there were no capacity limits preventing race and coalition from being retrieved at similar levels. Therefore, the sharp decline in racial categorization cannot by attributed to limits on a common pool of attention or working memory.

Other counter-hypotheses remain, however. Although gender categorization was high in both conditions, it did decrease significantly when coalitions were made visually-distinct. Regression toward the mean could account for this decrease—unlike race, categorization by sex was near ceiling in the gray condition (and higher than in most studies). This decline could be a real effect, however: It could be the product of a perceptual categorization system that has nothing to do with people, let alone alliances [29].

Perceptions of similarity can change when two visually-distinct categories are crossed [30], [31]. Compared to a situation in which gender is the only visually-distinct category, men and women wearing the same color may be confused more often, and same-sex individuals wearing different colors may be confused less often. The change in relative error rates will register as a decrease in gender categorization. This raises the possibility that the decrease in racial categorization had nothing to do with making cross-cutting alliances easier to track. It could reflect nothing more than a reduction in categorization by one visual difference (race) when it is crossed with another visual difference (shirt color). This possibility has never been directly tested, but will be in Studies 1 and 2 below.

Studies 1 and 2 have a second, more important function: They measure racial and gender categorization when coalitions are absent. Our strategy is to compare categorization by race and gender when coalitions are *absent* to visually-identical conditions in which coalitions are *present*. With this experimental design, coalition-induced decreases in racial (or gender) categorization can be detected without introducing visual distinctions. The effects of introducing peaceful coalitions are tested directly, using scenarios in which a discussion of mutually cooperative behaviors provides the only basis for inferring who is allied with whom.

## The Present Research

Ancestrally as now, people formed cooperative alliances for many different purposes, and who is allied with whom shifts depending on what common goal they are working toward—defeating a common enemy, providing food or shelter, helping someone in need [32]–[35]. What unites these situations is that in each, a set of individuals is cooperating to achieve a common goal. What distinguishes them is the common goal—different sets of allies will be relevant depending on whether one is organizing a rumble, a hunt, or an injured friend's care. For this reason, a well-designed alliance detection system should be activated by cues that several individuals are disposed to cooperate with one another, whether their common goal is peaceful or antagonistic.

To test this prediction, we created coalitional scenarios in which members of two charity groups are conversing in a friendly, non-competitive way about how each group cooperates to either build houses (*Habitat for Humanity*) or provide nutritional support (*Partners in Health*). If peaceful coalitional cooperation falls within the scope of the alliance detection system, then this genial interaction will trigger the formation of charity-based alliance categories (Studies 3 and 4).

To test the alliance system's retrieval functions, charity group membership was crossed with either race (Study 3) or sex (Study 4). We compared measures of racial and gender categorization from these studies to matching studies in which coalitions were absent (Studies 1 and 2). When coalitions are absent, measures of racial categorization should reflect the alliance system's estimate of the prior probability that race predicts alliances (Study 1). The system should revise this prior probability downward, however, when coalitions are present and organizing the speakers' behavior, but race is not (Study 3). As a result, the retrieval of racial categories will be down-regulated, relative to levels found when coalitions are absent.

When coalitions were present, we varied the sentences presented at recall as a further test of the alliance system's retrieval functions. Sentences in one condition implied that membership in *Habitat* versus *Partners* was relevant to the speakers; sentences in the other implied that the speakers were more interested in charitable activities than differences in group membership. If an alliance system exists, situational cues implying that differences in coalition membership are relevant to the speakers should up-regulate categorization by coalition and down-regulate categorization by race.

If gender and race are regulated by different systems—and race is an alliance category—then coalitional variables should have a selective effect: Unlike race, gender categorization should be the same whether coalitions are present (Study 4) or absent (Study 2). As a further control, we checked whether categorization by race (or sex) is diminished simply by crossing it with a second category, one unrelated to alliances (Studies 1 and 2).

Importantly, the coalitions in Studies 3 and 4 were *not* visually-distinct categories—members of both charity groups were wearing identical gray shirts. In consequence, Studies 1–4 isolate the effect of coalitional variables, without visual distinctions as a potential confound. They answer the following questions.

1. *Does coalitional cooperation activate alliance detection, even in the absence of conflict?* If it does, there will be categorization by charity group in Studies 3 and 4. We can see if the effect sizes are similar to those found for the coalitional conflict scenario tested by Kurzban et al (Exps 1, 3), given that the subject population and photos of male faces were the same.
2. *Is antagonism between rival coalitions necessary to erase social boundaries?* When the conversation implies that charity-based alliances are organizing the speakers' behavior, but race does not predict these alliances, the alliance system should respond by up-regulating categorization by charity group and down-regulating categorization by race.
3. *Are the conditions necessary to “erase race” fragile—limited to cases in which team colors or insignias flag coalitional alliances, visually grouping people as race does?* Detecting alliances requires inferences about underlying mental states—dispositions to cooperate with some individuals more than others in a given situation. Cues revealing dispositions to form alliances can be implicit in the content of a conversation or in patterns of cooperation and competition. If race is categorized because it has acquired predictive validity as an alliance cue in our social ecology, then introducing behavioral evidence that race no longer predicts alliances should diminish its use—even when there are no visual distinctions marking coalition membership.
4. *Are alliances encoded promiscuously but retrieved selectively?* A well-designed alliance detection system would encode who is allied with whom whenever such information is available. Retrieval should be more selective, however. In real life, each individual participates in a number of different coalitions, forming alliances that shift across issues and contexts. Which set of allies is relevant—and should therefore be retrieved—varies dynamically with the situation. If every alliance category that applies to a person were retrieved with equal strength in every situation, decision-making systems would be swamped with irrelevant information.

Well-engineered computational systems prioritize information, delivering the right data to the right mechanism at the right time [36], [37]. For this reason, the alliance system should retrieve a particular category of allies more robustly when there are cues that this alliance category will help one anticipate and understand the reactions, responses, and interactions of people in the situation at hand. As an initial test of this, we varied the extent to which conversational alliance cues were present at recall, while holding the coalitional information presented at encoding constant. All else equal, we expect more categorization by coalition—and less categorization by race—when the sentences at recall suggest coalition membership is relevant to the speakers.

1. *By manipulating alliance-relevant variables, can we demonstrate a dissociation between categorization by race and categorization by sex?* By hypothesis, a number of different evolved programs give rise to social categorization, with the alliance detection system being just one. Categorization by sex is likely to be regulated by a different evolved system, which is specialized for that function. Gender organizes social relationships in all primate species [38] and across human cultures [2]. In the ancestral environments that shaped the design of our minds, knowing a person's gender supported inferences about their behavior in many different social contexts—courtship, long-term mateships, parenting, warriorship, even foraging roles (hunting versus gathering). For this reason, one would expect a person's sex to be spontaneously encoded and retrieved by many different systems—ones regulating motivations, decisions, and inferences in each of these social domains.

This gender categorization system may sometimes interact with the alliance detection system, but its operations should be largely independent of it [1], [2]. If racial categorization is produced by an alliance detection system, then the effects of social variables relevant to alliance detection should be selective: there should be situations in which they regulate the retrieval of people's race, but not their sex.

These five claims about alliance detection, race, and sex are tested most directly by Studies 1–4, where charity group membership is not marked by any visual distinction. We report the results of these “gray” conditions in the main text. The supplemental materials present the details of parallel studies in which the coalitions are visually-distinct, as in Kurzban et al. (i.e., “color” conditions, Studies 5 and 6). These results are briefly discussed in the main paper, with an eye to which effects—if any—are enhanced by adding these colorful visual flags.

## When coalitions are absent: Categorization by race (Study 1) and sex (Study 2)

An advantage of the WSW paradigm is that racial and gender categorization can be measured holding everything constant except the scenario (the introduction and sentences). In contrast to the coalitional scenario that is used in Studies 3 and 4 (below), Studies 1 and 2 use a scenario that provides no basis for inferring alliances. As a result, they measure categorization by race (Study 1) and sex (Study 2) when coalitions are absent.

These studies use the same photos as those in which coalitions are present. Accordingly, all the targets are wearing gray shirts (*gray conditions*). Study 1 measures racial categorization for male targets (1a) and, separately, for female targets (1b). Study 2 measures gender categorization, using the white male and female targets from Study 1.

Because the same gray-shirted targets are used in Studies 1 and 3, we will be able to compare racial categorization in the presence and absence of coalitions, while holding all else constant. If race is treated as an alliance category, then categorization by race should decrease in Study 3, where it is crossed with charity group membership. Visual distinctions marking coalition membership should not be necessary to elicit this pattern.

Finding this pattern would not imply the operation of an alliance system, however, if racial categorization is diminished whenever race is crossed with a second category. To provide a strong test of this possibility, we crossed race (and sex) with a second category that is highly detectable, but has nothing to do with alliances: bright red versus bright yellow shirts. These *color conditions* were identical to the gray ones in every other way. Comparing racial categorization in the gray versus color conditions of Study 1 will reveal whether the introduction of a cross-cutting visual difference is sufficient to diminish racial categorization, even when it is not an alliance cue.

Introducing a visually-striking difference in color provides an apt test because it speaks to the long-held view that shared features and perceived similarity bootstrap the acquisition and retrieval of categories, whether the stimuli being classified are people or objects [14], [39]–[42]. Faces can be sorted by race and sex on the basis of shared visual features; these create within-category similarities and between-category differences in appearance that are easy to discern and always present. These perceptual similarities and differences have been thought to account, in part, for the fact that these social categories are easy to acquire, robustly retrieved, and difficult to extinguish by changing the social context [14], [19], [43], [44]. If similarity-driven processes do regulate the construction and retrieval of social categories, then it would be reasonable to expect the color conditions to (i) elicit the formation of visually-based categories, such as “red-shirted people” versus “yellow-shirted people”, and (ii) decrease categorization by race and sex. Similarity-based views predict a reduction in racial categorization because crossing race with shirt color should decrease the perceived similarity of same-race targets and increase the similarity of cross-race targets [30], [31]. They predict a similar decrease for gender categorization.

## Method

### Ethics Statement

All of the research reported herein was approved by the Human Subjects Committee at the University of California, Santa Barbara. Written informed consent was obtained from all participants.

### Participants

In this and all other studies reported herein, the participants were undergraduates at the University of California, Santa Barbara (UCSB), who were randomly assigned to conditions. They were recruited from an undergraduate population that is approximately 51% white, 21% Hispanic, 18% Asian, and 3% black (∼7% other/unknown). Some participants were enrolled in introductory psychology or anthropology classes and participated in exchange for partial course credit; others responded to a subject recruitment website that reaches students across departments and participated in exchange for pay.

In Study 1, there were 284 participants (141 females, 143 males; mean age 19.06, SD  = 1.74); 139 (68 female) in the gray shirt conditions, 145 (73 female) in the color shirt conditions. In Study 2, there were 136 participants (71 female), mean age 20.95 (SD 4.49); 68 (35 female) in the gray condition and 68 (36 female) in the color condition.

### Design

#### Study 1a—Racial categorization for male targets

There were two between-subjects conditions (gray vs. color). In each condition, there were eight male targets, four black and four white. In the *gray condition*, all eight targets were dressed in the same gray t-shirts. In the *color* condition, four targets were dressed in bright yellow t-shirts and four were dressed in bright red t-shirts. In the color conditions, race was crossed with shirt color: two black and two white targets wore red shirts, two black and two white targets wore yellow shirts.

#### Study 1b—Racial categorization for female targets

This study was identical to Study 1a, except all eight targets were female.

#### Study 2—Gender categorization

In Study 2, the targets were four men and four women (all white). Otherwise, its design was the same as for Studies 1a and 1b.

Each participant was randomly assigned to one of these six conditions.

### Materials

In Study 1, the targets were eight men (or eight women), four black and four white. Each was represented by a high resolution, torso length color photograph. All were wearing t-shirts and had a neutral expression. Photos in the gray and color conditions were identical except for shirt color. Photoshop was used to change shirts from their original gray color to red or yellow. Female targets had either short or tied-back hair. In Study 2, the eight targets were the four white men from Study 1a and the four white women from Study 1b.

### Procedure

Study 1 and 2 followed the same procedure; only the photos differed. Each participant was seated at a computer, in a laboratory equipped with separate, semi-private computer cubicles. Each session was assigned to either a gray or color condition, and the number of participants in each session could range from one to ten. After filling out consent forms and indicating their sex, participants read a short introductory story, which was presented on successive screens on the computer terminal.

This introduction said that a government program had randomly, yet extensively, sampled people from the U.S. population for research and data-collection purposes. In addition to answering surveys, these individuals were photographed and interviewed; participants were told that they would be seeing excerpts from the transcripts of these interviews, each paired with the interviewee's photo. They were told that we were interested in their impressions of these individuals. After this, photos of the targets (the interviewees) were shown sequentially, each paired with a statement that individual had made.

Each of the eight targets was shown three times; each time, a different excerpt from his or her interview was presented below his or her photo. The 24 statements (3 for each of 8 targets) were presented in the same sequential order, each for 15 seconds. The statements ranged from 30 to 91 words in length, and had content that was similar to the statements used in the coalitional conditions of Studies 3 and 4 (e.g., “We do have to do a lot of planning. We usually have specific plans before we start. It can be long and tedious, and can take weeks if not months. I promise it's not easy. We have to go over where we're going to be, make some kind of plan, buy supplies, and even worry about things like roads.” See File S1). The introduction explained that the targets were not talking to one another. Importantly, the content of the statements did not imply that any of the targets were allies, or belonged to the same coalition.

Although the 24 statements always appeared in the same order, which targets were associated with which statements was randomized across participants. There was one constraint on randomization: the first two speakers were always black, and the third and fourth speakers were always white (the rationale for this presentation order will be explained in Study 3). For the other 20 statements, the race and identity of targets was completely randomized.

In the color conditions, each participant saw a given target in either a red or a yellow shirt; the pairing of target with shirt color was randomized across participants. Shirt color alternated with every statement; the first target always wore a red shirt, the second a yellow shirt, the third a red shirt, and so on (to match Studies 5 and 6, where coalition is marked by shirt color; see SOM). The first four photographs always represented all four possible combinations of race and shirt color: black target in red shirt, black target in yellow shirt, white target in red shirt, white target in yellow shirt, in that order. Thereafter, assignment of targets to statements was randomized (with alternating shirt color the only constraint).

After viewing the six-minute statement presentation, participants were presented with a one minute distracter task in which they were asked to remember as many state capitals as possible with the aid of a U.S. map presented on screen. This distracter task, a standard part of the WSW paradigm, exists to reduce rehearsal and recency effects.

Immediately after the distracter task, the photos of all 8 targets appeared in an array on the screen. At this point, participants were told that they would be seeing the statements made previously, and would be asked to recall who said each statement by clicking on one of the eight photos. The 24 statements were then presented mid-screen in a random order. The placement in the array of the eight photos was constant for each participant, but randomized across participants. After attributing each of the 24 statements to a target, participants filled out several surveys. They were then debriefed and thanked.

### Categorization measures

Categorization measures were the same across all studies, so we will describe them once here, using as an example the conditions in which race was crossed with shirt color.

When a participant correctly identifies which target made a statement, this reveals nothing about categorization: They could have answered correctly by retrieving an accurate episodic memory, making a lucky guess, or by retrieving the speaker's race, sex, or other category memberships to make an informed guess. WSW paradigms are designed to elicit recall errors—responses in which the statement is attributed to the wrong speaker—because these are necessary to infer categorization. Fortunately, it is difficult to correctly recall who said what when there are 24 statements made by 8 targets; in Study 1, for example, the error rate was 70%, which is high, but better than chance (87.5%).

In a WSW paradigm with 8 targets differing in two crossed dimensions—race and shirt color—a participant can misattribute the speaker's statement to one of 7 other targets. These 7 fall into 4 categories with respect to the speaker: *same race, same* (shirt) *colo*r (*srsc*, 1 target), same race, different shirt color (*srdc*, 2 targets), different race, same shirt color (*drsc*, 2 targets), and different race, different shirt color (*drdc*, 2 targets). Because there are twice as many targets in the last three categories as in the first, the probability of making an *srsc* error by chance alone is half that of making each of the other errors. To mathematically correct for this difference in base rates, the other three error rates (*srdc*, *drsc*, *drdc*) are divided in half for each participant before analyses are carried out.

To measure racial categorization, a difference score is calculated for each subject: the number of same-race errors (choosing targets of the same race as the speaker) minus the number of different-race errors (choosing targets of a different race than the speaker), that is, [(*SR*sc + *SR*dc) − (*DR*sc+*DR*dc)]. The mean of these difference scores is compared to zero, which is the expected value if responses are random with respect to race, using a paired *t*-test. A mean difference significantly greater than zero indicates that participants made more same-race confusions than different-race confusions; it means that subjects both encoded the speaker's race, and retrieved it at recall. The effect size, *r*, associated with the *t*-test indicates how strongly the participants categorized by race; it can range from 0 to 1, with larger values indicating a greater tendency to make same-race than different-race confusions.

Categorization by shirt color is measured by an analogous difference score: the number of same-shirt color errors (choosing targets wearing the same shirt color as the speaker) minus the number of different-shirt color errors (choosing targets wearing a different shirt color than the speaker): that is, [(sr*SC* + dr*SC*) − (sr*DC* + dr*DC*)]. The rest of the analysis proceeds as described above, with the effect size, *r*, indicating how strongly the participants categorized targets by their shirt color.

A single condition in which the targets vary in two crossed dimensions will therefore yield two effect sizes, one for each dimension. For example, by comparing the effect sizes for race and shirt color in a single condition, one can see whether the participants encoded and retrieved one of these categories more than the other. By comparing effect sizes for race across conditions, one can see whether race is categorized more strongly under some circumstances than others.

Studies 1–6 involve 16 conditions; in 13 of them, the targets vary in two crossed dimensions (e.g., race and coalition, sex and coalition). In the remaining three (all gray conditions), there is only one dimension of variation, either race (Study 1a, 1b) or sex (Study 2). These are baseline conditions, in which all targets are wearing gray shirts and their statements provide no basis for inferring coalitional alliances. To correct for base rates in these conditions, we crossed race with a dummy variable that was randomly assigned to the targets. This allowed us to calculate categorization scores using the same base rate correction method as in the 13 conditions with two crossed dimensions.

For each categorization score, we report the size of the effect (*r*) and its significance (with *df*) in the main text, as these are the statistics most relevant to the hypotheses (within rounding error, *t* =  sqrt[(*r* ^2^*df)/(1−*r* ^2^)]). For those interested, the *t*-value from which each *r* was computed, along with means and standard deviations for the same-category errors, the different-category errors, and the difference scores, are reported in Tables S1 – S4 in File S1. All *p*-values are two-tailed. Every condition was checked to see if the results were qualified by sex of participant (every condition has ∼30 participants of each sex). Every linear regression comparing categorization across conditions controlled for sex of participant. Statistics for sex of participant tests are reported only when significant effects were found (there was only one such case). Data reported in this paper are available upon request to the first author.

## Results for Studies 1 and 2

**Figure 1:**
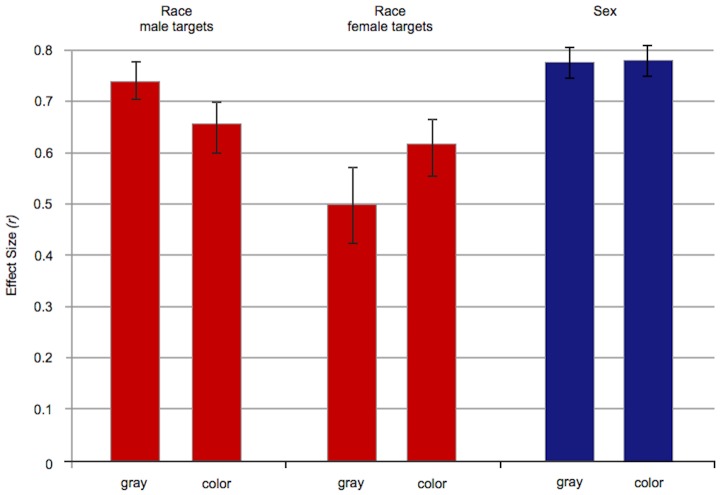
When coalitions are absent: Effect sizes for categorization by race (Study 1) and gender (Study 2). Racial categorization was same when race was the only visually distinct category (gray conditions) and when it was crossed with a vivid difference in shirt colors (color conditions). The same was true for gender categorization. Crossing race with a second category—one unrelated to coalitions—does not decrease racial categorization. (The same is true for gender categorization.) Error bars: +/− 1 S.E. of mean error difference.

When coalitions were absent, race and sex were categorized strongly in both the gray and the color conditions (see Figure 1). Despite the visual salience of red versus yellow shirts in the photos, racial categorization was not decreased by crossing race with shirt color (gray vs. color conditions). The same was true for categorization by sex. (None of these results were qualified by sex of participant.) More specifically:

### Study 1: Racial categorization without coalitions

When all targets were wearing identical gray shirts, participants made more same-race errors than different-race errors; this was true whether they were presented with male targets (Study 1a: *p* = 10^−13^, *df* 70) or female targets (Study 1b: *p* = 10^−5^, *df* 67). The effect sizes for race were large: *r* = .74 for male targets and *r* = .50 for female targets.

#### Does merely crossing race with a second category reduce racial categorization— even when the second category has nothing to do with coalition membership?

No. In the color conditions, race was crossed with a very obvious visual distinction, which divided the targets into two equal sized categories: red-shirted people versus yellow-shirted people. Nevertheless, racial categorization remained strong, with an effect size of *r* = .66 for male targets (*p* = 10^−9^, *df* 68) and *r* = .62 for female targets (*p* = 10^−8^, *df* 75).

Crossing race with shirt color did not reduce racial categorization from the levels found when race was the only dimension of variation (the gray conditions). Linear regression shows that levels of racial categorization in the color conditions were not lower (or higher) than those found in the gray conditions, whether the targets were male (*t*(137)  = 0.75, *p* = .45, *r* = .06) or female (*t* (141) = −0.60, *p* = .55, *r* = −.05).

#### Was racial categorization the same for male and female targets?

Hunter-gatherer societies have a sexual division of labor, and several important domains of coalitional cooperation are associated more with men than women (e.g., warfare, cooperative hunting, cooperative shelter-building). This raises the possibility that alliance-tracking systems are more easily activated by male than female targets [2]. If race is encoded as a coalition cue, this might lead one to expect that people will be more likely to categorize male than female targets by their race.

A linear regression (collapsing across the gray and color conditions, which did not differ) showed that race was categorized at slightly higher levels for male than female targets (*p* = .005, *r* = .16 [combined *M*_diff_ (SD): 2.69 (2.74) for male targets, 1.81 (2.70) for female targets, *t*(280) = 2.81]). As always, this analysis controls for sex of participant (no effect).

### Study 2: Gender categorization without coalitions

Participants made more same-sex errors than different-sex errors when all the targets were wearing gray (*p* = 10^−14^, *df* 67); the same was true when red versus yellow shirt color was crossed with sex (*p* = 10^−14^, *df* 67). In both cases, the effect size for gender categorization was large: *r* = .78.

Crossing sex with a difference in shirt color did not reduce categorization by sex from the level found in the gray condition (linear regression: *t*(133) = 0.73, *p* = .47, *r* = .06; no effect of participant gender).

#### Is categorization by race and sex just a byproduct of how the perceptual system works? Measuring categorization by shirt color when it does not signify coalitional alliances (see Tables S2, S3 in File S1)

It is tempting to think that people strongly categorize others by their race and sex because our perceptual system cannot do otherwise. Most adult faces can be categorized as *black* or *white*, or as *male* or *female*, on the basis of visual features alone. Does the visual system register similarities and differences in the visual features of *any* set of objects, and automatically sort them into categories on the basis of the patterns it detects? If this were true, then categorization by race and sex would occur simply as a byproduct of how the perceptual system works.

The color conditions of Studies 1 and 2 allow a straightforward test of this hypothesis. The visual difference between bright red and bright yellow shirts is perceptually salient in these conditions, and it exhaustively classifies the targets into two equal sized categories: *red-shirted people* and *yellow-shirted people*. If visual similarities and differences are sufficient to elicit the formation of social categories, then these conditions should consistently elicit strong categorization by shirt color.

They did not. When shirt color was crossed with sex (Study 2), participants did not categorize the targets by their shirt color at all (*r* = .02); they made as many same-color errors as different-color errors (*p* = .85, *df* 67). This failure to categorize by shirt color is not because constraints on attention force a tradeoff between two crossed dimensions. As Kurzban et al. demonstrates (and Study 4 replicates), participants can simultaneously categorize targets by two crossed dimensions—gender and coalition membership—at very high levels.

The results were similar in Study 1, where shirt color was crossed with race. Female targets were not categorized by their shirt color to a significant extent (Study 1b: *p* = .29, *df* 75, *r* = .12). When viewing male targets, participants did make more same-color than different-color errors (Study 1a: *p* = .026, *df* 68, *r* = .27), but a linear regression showed that this effect was fully accounted for by sex of participant (sex effect: *r* = .24, *p* = .047, *t*(67) = 2.02). Controlling for this variable dropped the remaining categorization average to zero (*r* = .013, *p* = .92, *t*(67) = 0.11). This was the only case (out of 33 possibilities herein) in which a categorization score was qualified by the sex of participants.

Nevertheless, we followed up by analyzing each sex separately for Studies 1a and 1b. This revealed a consistent pattern. Both women and men categorized opposite-sex targets by their shirt color to a moderate extent: *r* = .41 for women viewing men (1a: *p* = .01, *df* 36), and *r* = .33 for men viewing women (1b: *p* = .037, *df* 39)—an effect that may reflect greater interest in the opposite sex among young, mostly unmarried, adults. By contrast, neither men nor women categorized same-sex targets by their shirt color at all (for men (1a): *p* = .90, *df* 31, *r* = 0.02; for women (1b): *p* = .36, *df* 36, *r* = − .15). If later research shows that this pattern is real, it would not change the basic finding: The fact that shirt color categorization disappears for same-sex targets (and did not appear at all when shirt color was crossed with sex) shows that visual similarities and differences are not sufficient to elicit the formation of social categories.

### Summary: What do Studies 1 and 2 show?

#### Baseline measures

Studies 1 and 2 measured racial and gender categorization in the absence of coalitions. When given no alternative basis for inferring alliances, participants strongly categorized targets by their race and their sex, with large effect sizes. The gray conditions of Study 1 provide baseline measures of racial categorization; we use these to determine whether racial categorization is reduced by the introduction of cross-cutting coalitions in Study 3. The gray condition of Study 2 provides the baseline measure of gender categorization for Study 4, which crosses coalition with sex.

#### Crossing categories is not enough

If an alliance detection system regulates categorization by race but not sex, then the effects of crossing coalition membership with these social categories will be selective: It will down-regulate categorization by race but not sex. In this light, it is important to make sure that racial categorization is not down-regulated whenever race is crossed with a second category. To provide a strong test of this possibility, we crossed race with a striking visual difference that exhaustively classified the targets: red versus yellow shirts. Crossing race with shirt color did not decrease racial categorization from the levels found when race was the only dimension of variation (the gray conditions).

This conclusion is not qualified by how strongly participants categorized the targets by their shirt color in Studies 1a and 1b. The same result—no decrease in racial categorization from gray to color conditions—obtains even when the analysis is restricted to opposite-sex targets, who were categorized by shirt color to a moderate extent. (Men categorizing female targets by race: *p* = .51, *r* = −.08 [*M*_diff_ (SD): gray  = 1.99 (2.87), color  = 2.38 (2.25), *t*(73)  = −0.66]. Women categorizing male targets by race: *p* = .66, *r* = .05 [M_diff_ (SD): gray  = 2.83 (2.32), color  = 2.54 (3.08), *t*(70)  = 0.45]).

Because Study 1 directly compared crossed and uncrossed conditions that were otherwise identical, it is the first WSW study to show that racial categorization is not diminished by crossing race with a second category that is highly salient and purely perceptual. It also sheds new light on the coalitional conflict studies by Kurzban et al. In support of the argument that race is treated as a coalitional cue, they showed that racial categorization decreased when membership in rival coalitions was marked by differences in shirt color. However, a skeptic could have argued that this reduction in racial categorization reflected nothing more than a reduction in categorization by one visual difference (race) when it is crossed with another visual difference (shirt color). The results of Study 1a and 1b clearly undermine this non-coalitional interpretation: Introducing a difference in shirt color that does not mark coalition membership failed to reduce racial categorization. Indeed, Study 1a demonstrated this using the same male faces and subject population tested by Kurzban et al. (Exps 1 & 2).

#### Evaluating the perceptual byproduct hypothesis

Visual similarities and differences are not sufficient to elicit the formation of social categories. Instead of finding consistently strong categorization by shirt color in Studies 1 and 2, participants did not categorize by shirt color at all in two cases, and did so only moderately in the third. This is a typical result: Past findings also support the claim that purely visual distinctions elicit little or no categorization in the WSW. In a design similar to Study 1b with female targets (and a female-biased sample of participants), Stangor et al. (Exp 5 [19]) found strong categorization by race but no categorization by shirt color. The same is true for visual differences that, like skin color, are intrinsic to the body, but have nothing to do with race. In Sack [45], half the targets had a large wine-stain birth mark on their faces and the other half did not. The context attributed the wine-stain birth mark to a gene that was prevalent in the population, but had no other effects. This very obvious difference in physical appearance did not elicit *any* categorization. Yet the very same visual distinction elicited strong categorization when the context said it marked the targets' social alliances.

Categorization by race and sex cannot be a byproduct of a perceptual system that automatically sorts individuals into categories based on purely visual features (ones with no social meaning), because these results show that the visual system does not work that way—at least when the stimuli are people.

#### Categorization is not inevitable

The shirt color results also underline an important methodological point. A reasonable person might wonder if any difference that cleanly divides eight people into two equal sized groups will elicit the formation of social categories in a WSW study—either as a demand characteristic or because the perceptual system cannot do otherwise. But the shirt color results show that this is not true. In two out of three cases, there was no evidence that participants categorized the targets as “red-shirted people” and “yellow-shirted people”, even though shirt color was a simple, obvious visual feature that cleanly divided eight people into two equal sized groups. The same result has been found using behavioral, rather than visual, categories [3], [17]. This implies that categorization by race, sex, and coalition is not an artifact of the experimental design or a side-effect of how the visual system works.

With these points in mind, we now turn to Studies 3 and 4, which cross race and sex with coalition membership.

## When coalitions are present: Crossing coalition membership with race (Study 3) and sex (Study 4)

### Study 3: Categorization by coalition and race

The purpose of Study 3 was to investigate the conditions that activate the alliance detection system, and see how this affects categorization by race. Study 3 was identical to Study 1, except for the text provided—the introductory story and statements made by targets. The text for Study 1 provided no coalitional information—nothing to suggest that each target belonged to one of two coalitions. In contrast, the story and statements for Study 3 implied that each target belonged to one of two charity groups, each composed of individuals who coordinate and cooperate with one another to achieve a common goal—helping people.

Members of both charity groups were identically dressed in gray shirts, so there was no visual feature distinguishing them. The only cues from which alliances could be inferred were behavioral—the comments made by each target about their coalitional activities. These behavioral cues were always present during the encoding phase in Study 3, but we varied the recall context. In the *coalitions-relevant* conditions, the sentences presented at recall suggested that charity group membership was relevant to the speakers. In the *coalitions-irrelevant* conditions, the sentences presented at recall suggested that charitable work, but not coalition membership per se, was relevant to the speakers.

We have proposed that the alliance detection system encodes and stores information about who is allied with whom when it is available, but retrieves this information selectively, in response to cues indicating which alliance categories are most relevant to the situation at hand. This leads to the prediction that charity group membership will be encoded and stored across conditions, but it will be retrieved most strongly when the recall context suggests that it is relevant to the targets. If this is correct, then scores indexing coalitional categorization will be stronger when the sentences at recall are coalition-relevant than when they are coalition-irrelevant.

In Study 3, coalition membership was crossed with race, such that each charity group had two black and two white members. Thus race did not predict charity-based alliances. We will compare levels of racial categorization in these conditions with the baselines established in Study 1, where there were no coalitions. The hypothesis that the alliance system regulates categorization by race predicts that the same cues that up-regulate categorization by charity group will down-regulate categorization by race. This implies that the most dramatic reductions in racial categorization will be found when the recall context implies that charity group membership is relevant to the targets.

### Study 4: Categorization by coalition and sex

Study 4 was identical to Study 3, with one exception: coalition membership was crossed with sex, not race. That is, each charity group had two male and two female members, all of whom were wearing gray shirts.

Coalitional categorization in Study 4 should follow the same pattern as in Study 3. Gender categorization should not. By hypothesis, gender categorization is regulated by a system that is functionally distinct from the alliance detection system. In consequence, cues that up-regulate categorization by charity group should have no effect on categorization by sex. This will be tested by comparing levels of gender categorization in Study 4 with the baselines established in Study 2, where there were no coalitions.

## Method

### Participants

Study 3 (coalition x race) had 286 participants (158 female), mean age 19.35 (SD 2.26). Study 4 (coalition x sex) had 138 participants (67 female), mean age 19.93 (SD 3.81).

### Design for Study 3

There were two between-subjects conditions involving male targets (Study 3a), one in which the sentences at recall were coalition-relevant and one in which they were coalition-irrelevant. To see if the results generalize to coalitions composed of women, the same two between-subjects conditions were conducted using female targets (Study 3b). Participants were randomly assigned to conditions.

In every condition, the eight targets were of the same sex and identically dressed in gray shirts. Because there were no visual cues to coalition membership at encoding or at recall, structural fit inferences [25], [46]–[48] are impossible; they cannot influence measures of coalitional categorization in this or any other gray-shirt condition (see SOM for discussion). Structural fit inferences cannot influence measures of categorization by race or gender either; although these are visually-distinct categories, none of the sentences have content relevant to inferring race or gender.

Race was crossed with charity group membership, such that each charity group was composed of two black and two white members. Verbal cues to coalition membership were always present at encoding—that is, during the targets' conversation. During this conversation, each statement made by a target included sentences that would allow one to infer his coalition membership (*coalition-relevant* sentences), alongside sentences that could have been said by members of either coalition (*coalition-irrelevant* sentences).

Although verbal cues to coalition membership were always present at encoding, conditions differed in whether such cues were present at recall. When participants were asked to recall who said what, the sentences they were asked about contained verbal cues to coalition membership in the *coalitions-relevant* conditions, but not in the *coalitions-irrelevant* conditions. This manipulation allowed us to address questions about the role of encoding versus retrieval in coalitional categorization, and how each affects the retrieval of race.

### Materials and Procedure for Study 3

The procedure for Study 3a (male targets) was identical to that for Study 1a. Study 3a differed only in the short introductory story and the content of the statements (SOM). At encoding, the word length of each statement was identical to Study 1.

The introductory story for 3a explained that participants would be seeing a conversation among members of two different volunteer-based charitable organizations, each dedicated to solving a different problem faced by poor people. Volunteers for *Partners in Health* work to eradicate hunger by improving nutrition and agricultural practices; volunteers for *Habitat for Humanity* build homes for people living in poverty. Although both groups had been working in nearby rural areas, their members had not met until they found themselves traveling on the same bus. Participants were told that the ensuing conversation had been recorded and they would be seeing a portion of it. They were told we were interested in their impressions of these volunteers.

The target photos were identical to those used in Study 1a. The computer program assigned two black and two white targets to each charity group, randomizing the assignment of targets across participants. Thus the same target individual was a member of *Habitat* for some participants and a member of *Partners* for others.

As in Study 1, 24 statements were presented in the same sequential order, each for 15 seconds. Each of the eight targets was paired with three statements; thus 12 statements were contributed by each charity group (4 targets per group x 3 statements). A portion of each target's statement contained information from which one could infer his or her coalition membership.

The first statement was made by a member of *Habitat for Humanity*, the second by a member of *Partners in Health*, and so on, with the conversation alternating back and forth between the two coalitions. Across participants, targets were randomly assigned to the statements made by their group members.

This random assignment was subject to one constraint: the first two speakers were always black, and the third and fourth speakers were always white. Thus the order of the first four speakers was always black *Habitat*, black *Partners*, white *Habitat*, and white *Partners*. This presentation order was designed to (i) activate race at the outset of the conversation, while (ii) providing early evidence that race does not predict coalition membership in this situation. Early activation of race as a category increases the chance that participants will notice that race does not predict charity group membership in this short conversation; having two black speakers start the conversation was more likely to have this effect, given that most of the participants were white, Hispanic, and Asian. At the same time, this presentation order ensured that race and coalition were uncorrelated for the first four statements: same-race targets belonged to different coalitions, and same-coalition targets were a different race. For the 20 statements that followed these first four, the order of the targets (including their race) was random across participants.

After viewing this six-minute conversation, participants were presented with the same one minute distracter task as in Study 1 (thinking of state capitals). Immediately afterward, they were presented with an array of all eight target photos. They were told that they would be seeing a portion of the statements made previously, and would be asked to recall who said each statement by clicking on one of the eight photos. At recall, the order of presentation for the 24 statements was randomized across participants, as was the order of photos in the array.

#### Content of statements at encoding

The introductory story and statements made during the conversation are presented in File S1. Because the introductory story explained what each charity does, statements during the conversation that mention activities and equipment relevant to building houses support the inference that the target belongs to *Habitat for Humanity*, and statements relevant to planting crops and providing nutritional supplements imply that the target belongs to *Partners in Health*.

Each statement made by a target during the conversation contained sentences that would allow one to infer his coalition membership (*coalition-relevant* sentences) along with sentences that could have been said by members of either coalition (*coalition-irrelevant* sentences). For example, a target could say:

“*Some of us are nutritionists, some trained in agriculture. Some of us are specifically trained on the computer programs and the instruments for measuring soil quality and nutrition levels.* We've all had to have some minimal level of training. We work together on the big general projects, and then based on our backgrounds and experience we also each specialize.” (No italics in the stimuli.)

From the first two sentences, one can infer that this individual belongs to *Partners in Health*, and

is therefore in a cooperative alliance with other members of that charity group. We refer to these sentences as *coalition-relevant* because they can be used to infer the target's coalition membership and, therefore, who his allies are.

In contrast, the second two sentences could have been said by members of either charity group. When considered in isolation, they provide no information about the target's coalitional affiliation and alliances. For this reason, we refer to these sentences as *coalition-irrelevant*—they are irrelevant to distinguishing targets by their coalition membership.

The coalition-relevant sentences came first in 11 of the 24 statements, and their length ranged from 11–54 words; coalition-irrelevant sentences came first in the other 13 statements, and their length ranged from 17–54 words. (In the statement above, for example, a 29 word coalition-relevant portion is followed by a 30 word coalition-irrelevant portion.)

#### Content of statements at recall

When asked to recall who said what, participants were presented with a portion of each of the 24 statements; which portion they saw depended on the condition to which they had been assigned.

In the *coalitions-relevant* conditions, the only sentences presented at recall were those relevant to inferring the target's coalition membership. Every one of these sentences refers to activities that distinguish the coalitions from one another. When presented at recall, this set of sentences suggests that charity group membership is important to the speakers.

In the *coalitions-irrelevant* conditions, the only sentences presented at recall were those that could not, when seen in isolation, be used to infer the target's coalition membership. These sentences refer to charitable activities, but do not distinguish the speakers by charity group; indeed, each of them could have been said by any of the eight targets. Presenting this set of sentences at recall suggests that charitable activities are important to the speakers, but distinguishing individuals by their group membership is not.

The conversation had included both types of sentences; presenting only one type at recall should shift perceptions of conversational relevance [49], from charitable activities in general (*coalition-irrelevant* conditions) to distinguishing people based on their alliances (*coalition-relevant* conditions). If alliances are encoded promiscuously but retrieved selectively, then this shift in relevance should elicit differences in the extent to which charity group membership—and race—are retrieved in these two conditions.

Study 3b was identical to 3a, except it used the same female targets as Study 1b.

### Design, Materials, and Procedure for Study 4

Study 4 was identical to the male target conditions of Study 3, with a single exception: the targets differed in sex rather than race. As a result, Study 4 measures categorization when sex is crossed with charity group membership. The photos were the same ones used in Study 2.

## Results and Discussion for Studies 3 and 4

### Coalitional categorization, Studies 3 and 4 (see Table S2 in File S1)

**Figure 2:**
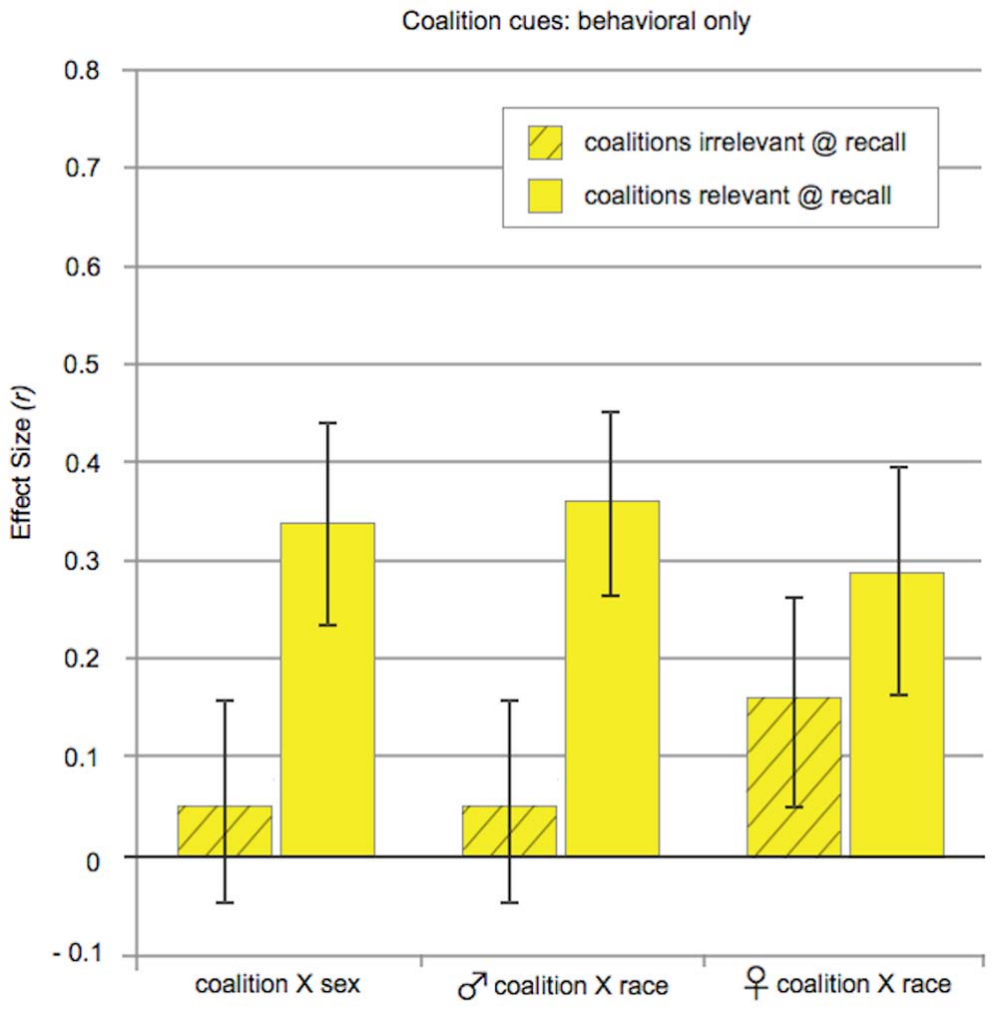
Effect sizes for categorization by coalition (Studies 3 and 4). Targets in these conditions all wore gray shirts, so the only cues to coalition membership were behavioral. ♂, ♀ denotes sex of target stimuli. Coalitional categorization followed the same pattern, whether coalition was crossed with sex or race: Charity group membership was retrieved more strongly when the sentences at recall implied that differences in coalition membership were relevant to the speakers than when they did not (coalitions-relevant vs. coalitions-irrelevant conditions). Error bars: +/− 1 S.E.

Figure 2 presents effect sizes for categorization by coalition when the two coalitions are interacting peacefully. Because all targets were wearing gray, behavioral cues of cooperation provided the only basis for inferring coalition membership. The results were the same whether charity group membership was crossed with race (Study 3) or sex (Study 4), so we will discuss them together.

#### Is coalition membership categorized in the absence of conflict, based solely on behavioral cues

Yes. During the encoding phase of each study, behavioral cues to coalition membership were identical across conditions. All that varied was whether the sentences at recall were coalition-relevant or coalition-irrelevant. An alliance detection system that encodes coalitional information promiscuously but retrieves it selectively will produce a distinctive pattern. (i) Categorization by charity group will be elicited when the recall sentences are coalition-relevant, and (ii) this effect will be stronger than when the recall sentences are coalition-irrelevant. That is what happened.

#### Coalitions-relevant conditions

Coalition membership was encoded and retrieved in these conditions based on behavioral cues alone—there were no visual markers distinguishing who was allied with whom. When the sentences presented at recall were coalition-relevant, participants made more same-coalition errors than different-coalition errors. This was true whether charity group was crossed with race (Study 3a-male targets: *r* = .36, *p* = .002, *df* 71; Study 3b-female targets: *r* = .29, *p* = .027, *df* 58) or sex (Study 4: *r* = .34, *p* = .004, *df* 66). Indeed, the effect sizes were very similar across the three charity studies—*r*s  = .36, .29, .34.

In these conditions, each sentence at recall referred to activities specific to *Habitat* vs. *Partners*. Could these participants have chosen targets who belong to the coalition implied by the sentence *without* having accessed stored representations that pair these individuals with their coalition membership?

No. The photo arrays at recall presented all eight targets dressed identically in gray. As a result, there were no visual cues to coalition membership to which inferences made at recall could be matched (i.e., no “structural fit”). To systematically choose targets who belong to the coalition implied by the sentence, a participant making these inferences would have to access stored representations of the targets that *pair their faces with their coalition membership*. Tagging a person's identity—represented by their face, their actions, or their words—with an alliance category is precisely what it means to encode and store their membership in a coalition. The only way to generate a positive and significant score for coalitional categorization in these gray conditions is to retrieve representations of the targets that had been tagged with their coalition membership during the encoding phase.

#### Coalitions-irrelevant conditions

In these conditions, coalitional information was present at encoding, but the sentences presented during the recall phase could have been said by members of either coalition. They contained no content that (i) distinguished targets by their charity group membership or (ii) suggested that group membership mattered to them. Moreover, everyone was identically dressed, so there were no visual cues distinguishing targets by their group membership. As a result, the recall phase contained no cues, either verbal or visual, that differences in charity group membership are of interest to the targets. Under these circumstances, there was no evidence that participants retrieved the coalition membership of the targets: They made about the same number of same-coalition errors as different-coalition errors, whether charity group was crossed with race (male targets: *p* = .67, *r* = .05, *df* 74; female targets: *p* = .16, *r* = .16, *df* 79) or sex (*p* = .67, *r* = .05, *df* 70).

#### Were alliances encoded promiscuously but retrieved selectively, in response to cues that they are relevant to the unfolding situation

An alliance detection system should be designed to encode alliance information when it becomes available, for later use. Each person belongs to many different coalitional alliances, however, and only a few of these will be useful for interpreting any given situation. The immediate situation—the recall context—should regulate which alliance categories are retrieved. For this reason, the alliance detection system should be designed to respond sensitively to cues in the recall context, up-regulating the retrieval of those coalitional categories likely to be most useful in understanding the situation, and down-regulating the retrieval of other alliance categories.

If the alliance system has this property, then charity group membership will be retrieved most strongly (and race most weakly) when the recall context suggests that membership in *Habitat* versus *Partners* is relevant to the speakers

#### Selective retrieval

As predicted, retrieval of coalition membership was more robust when the sentences at recall were coalition-relevant than when they were coalition-irrelevant: The effect sizes were *r* = .36 vs. *r* = .05 in Study 3a (male targets), *r* = .29 vs. *r* = .16 in Study 3b (female targets), and *r* = .34 vs. *r* = .05 in Study 4. This was confirmed by a linear regression that controlled for study (3a, 3b, 4) and participant sex: There was a main effect of having coalition-relevant sentences at recall (*F*(1, 420)  = 8.67, *p* = .003, *r* = .14), but no effects due to study or participant sex, and no interactions. Linear regressions on each study separately demonstrated that the difference between the coalition-relevant and coalition-irrelevant conditions was significant for Study 4 (*t*(135)  = 1.99, *p* = .048, *r* = .17) and for the male targets of Study 3a (*t*(144)  = 2.00, *p* = .047, *r* = .16). For the female targets of Study 3b, the difference between the two conditions did not reach significance, but the pattern was the same—coalition was retrieved to a significant extent when the sentences at recall were coalition-relevant, but not when they were coalition-irrelevant (*t*(136) = 1.06, *p* = .29, *r* = .09).

This pattern supports the hypothesis that retrieval of coalitional alliance categories is selective: Charity group membership was retrieved more robustly when the recall context contained cues that differences in group membership were relevant than when it did not. This cannot be attributed to greater difficulty in attributing coalition-irrelevant sentences to individual targets: Although they did not elicit high levels *coalitional* categorization, these same sentences supported robust levels of racial categorization and high levels of gender categorization (see below).

#### Promiscuous encoding

All participants were exposed to the same behavioral cues to coalition membership during the encoding phase; this suggests that participants encoded information about the targets' coalition membership in all conditions, but only retrieved it when the sentences at recall were coalition-relevant. If the targets' alliances were encoded (but not retrieved) when the sentences at recall were coalition-irrelevant, then there could be evidence of this in the data on racial categorization—it might be lower than it was Study 1, where there were no cross-cutting alliances to encode. This effect was present in the data (see below).

#### Do charity groups interacting peacefully elicit as much coalitional categorization as coalitions in conflict

Because we did not compare peaceful to antagonistic coalitions within each study, our data do not address this question directly. But we note that the effect sizes for coalitional categorization in the analogous conflict studies by Kurzban et al. were r = .31 (Exp 1: male targets, coalition x race) and r = .35 (Exp 3: coalition x sex). These studies used coalition-relevant sentences at recall, the same subject population, and the same male faces as our charity studies (3a and 4). Categorization by charity group was surprisingly similar, with coalitional effect sizes of r = .36 (male targets: coalition x race) and r = .34 (coalition x sex).

### Gender categorization, Study 4 (see Table S4 in File S1)

**Figure 3:**
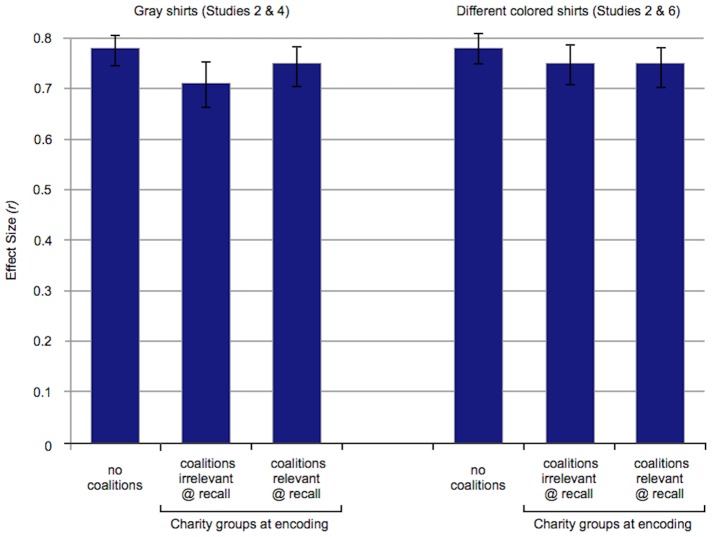
Gender categorization does not respond to coalitional variables (Studies 2, 4, and 6). The left panel depicts effect sizes for gender categorization when all targets were wearing gray shirts. The right panel depicts effect sizes for gender categorization when gender was crossed with shirt color. In each panel, the leftmost bar (labeled “no coalitions”) depicts gender categorization when coalitions were absent (Study 2). The two bars to its right depict gender categorization when coalitions were present and crossed with sex (Study 4: gray shirts; Study 6: different colored shirts). Gender was retrieved at the same high levels no matter how strong the coalitional cues; there were no significant differences between conditions. Error bars: +/− 1 S.E.

Figure 3 displays the effect sizes for gender categorization when coalitions were present (Study 4) and when coalitions were absent (Study 2, gray baseline).

#### Is categorization by sex reduced when peaceful coalitions interact, and sex does not predict who is allied with whom

No. In Study 2, we measured how strongly people categorized the same targets by their sex when there were no cross-cutting coalitions and everyone was identically dressed in gray (baseline gender categorization). Under these conditions, the effect size for categorization by sex was r = .78. Categorization by sex was virtually identical in Study 4, where sex was crossed with coalition membership.

#### Coalitions-relevant condition

Participants strongly categorized targets by their sex—*r* = .75—making more same-sex than different-sex errors (*p* = 10^−12^, *df* 66). Linear regression showed that this gender categorization score was no different from that found in the gray baseline condition, where there were no cross-cutting coalitions (*t*(132)  = 0.89, *p* = .37, *r* = .08).

#### Coalitions-irrelevant condition

Categorization by sex was strong in this condition as well—*r* = .71 (*p* = 10^−11^, *df* 70). Linear regressions confirm that this gender categorization score is not different from the gray baseline found in Study 2 (*t*(136) = 1.17, *p* = .24, *r* = .10) or in the coalitions-relevant condition of Study 4 (*t*(136) = 0.32, *p* = .75, *r* = .03).

### Racial categorization, Studies 3a and 3b (see Table S1 in File S1)

**Figure 4:**
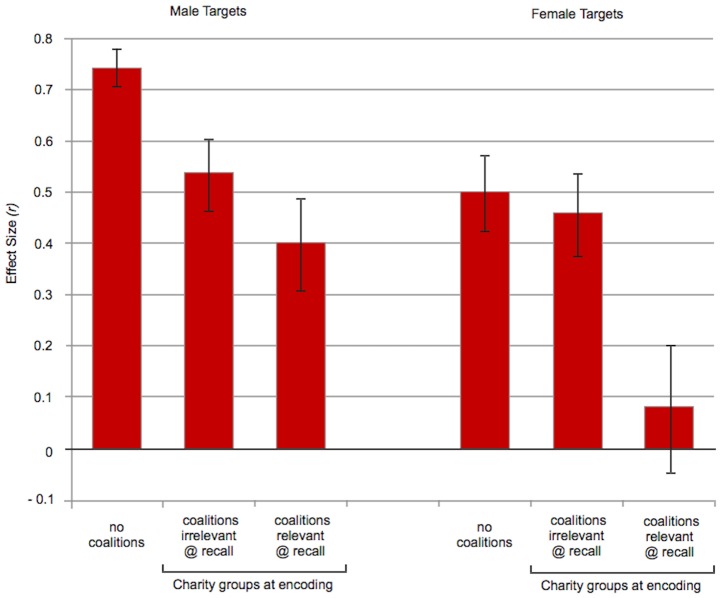
Coalitional variables regulate racial categorization (Studies 1 and 3). All targets wore gray shirts in these conditions; the only cues to charity group membership were behavioral. Effect sizes for racial categorization are shown for male targets (left panel) and female targets (right panel). In each panel, the leftmost bar (labeled “no coalitions”) depicts racial categorization when coalitions were absent (Study 1). The two bars to its right depict racial categorization when coalitions were present and crossed with race (Study 3). Behavioral cues to coalition membership were present at encoding for all the coalition conditions of Study 3. The sentences at recall varied across conditions: Their content was either relevant to coalition membership (“coalitions relevant @ recall”) or applicable to members of both coalitions (“coalitions irrelevant @ recall”). Retrieval of racial categories was down-regulated most strongly when the recall context included behavioral cues that coalition membership was relevant to the targets—the same recall contexts that up-regulated the retrieval of charity group membership. Indeed, race was “erased” (not retrieved to a significant extent) for female targets in this condition. Error bars: +/− 1 S.E.

Gender categorization did not respond to coalitional variables, but racial categorization did. Figure 4 displays the effect sizes for racial categorization when coalitions were present (Study 3) and when coalitions were absent (Study 1, gray baselines).

#### Is categorization by race reduced when peaceful coalitions interact, and race does not predict who is allied with whom

In Study 1, we measured how strongly people categorize the same targets by their race when there are no cross-cutting coalitions. When everyone in Study 1 was identically dressed in gray—as they were in Study 3—the effect size for categorization by race was r = .74 for male targets (Study 1a) and r = .50 for female targets (Study 1b). Was categorization by race lower in Study 3, where cross-cutting coalitions were present?

#### Coalitions-relevant conditions

Yes. When the sentences at recall implied that coalition membership was relevant to the targets, racial categorization was significantly lower than it was in Study 1, where there were no cross-cutting coalitions (linear regressions, male targets: *t*(140)  = 3.91, *p* = .0001, *r* = .31; female targets: *t*(124)  = 2.85, *p* = .005, *r* = .25).

When coalitions were present, just how strongly was race categorized? In Study 3, participants made more same-race than different-race errors for male targets (3a: *p* = .0004, *df* 71), but not for female targets (3b: *p* = .56, *df* 58). This means they retrieved the race of male targets to a moderate extent (*r* = .40), but they did not retrieve the race of female targets at all (*r* = .08).

This represents a striking decrease in categorization by race when cross-cutting coalitions were present compared to when they were absent—from *r* = .74 to *r* = .40 for male targets, and from *r* = .50 to *r* = .08 for female targets. Indeed, introducing cross-cutting coalitions led to race being “erased” for female targets: When the sentences at recall were coalition-relevant, participants did not retrieve these women's race at all.

In these conditions, information about the targets' coalitional alliances was present at both encoding and recall. What happens to racial categorization when coalitional information is present at encoding, but *not* at recall?

#### Coalitions-irrelevant conditions

When the sentences presented at recall were coalition-irrelevant, the effect sizes for race were *r* = .54 (male targets) and *r* = .46 (female targets). Participants made more same-race than different race errors for both male targets (3a: *p* = 10^−6^, *df* 74) and female targets (3b: *p* = 10^−5^, *df* 79).

In this condition, racial categorization for male targets was significantly lower than in Study 1a, where there were no cross-cutting coalitions to encode (*t*(143)  = 2.68, *p* = .008, *r* = .22). For female targets, there was not a significant decrease compared to Study 1b (*t*(145)  = 0.58, *p* = .56, *r* = .05), even though the effect size for race in the coalitions-irrelevant condition was descriptively lower for female than for male targets (*r* = .46 vs. .54). We note that racial categorization for female targets in Study 1b (*r* = .50) was lower than in any other baseline race condition (even the baseline color conditions of Study 1).

#### To reduce racial categorization, does coalition membership have to be retrieved or just encoded

The principle of promiscuous encoding with selective retrieval predicts that the recall context will affect retrieval of race *and* coalition. This was supported by the data. A recall context in which every sentence refers to coalition-specific activities suggests that a very relevant alliance category to retrieve is whether the target belongs to *Habitat for Humanity* or *Partners in Health* (the coalitions-relevant conditions). As predicted, these conditions elicited robust retrieval of coalition membership for male and female targets, along with the largest and most consistent drops in categorization by race.

Retrieval was selective: When the recall context made no mention of coalition-specific activities, and referred only to experiences and training common to both groups, coalition membership was not retrieved at all (the coalitions-irrelevant conditions). But were the targets' coalitional alliances encoded and stored in these conditions? It is reasonable to assume that they were, because their encoding contexts were identical to those in the coalitions-relevant conditions, where coalition membership was retrieved. Moreover, the effect on racial categorization for male targets provides indirect evidence for this interpretation. When coalitional information was present at encoding, but not retrieved, categorization by race was significantly lower than in Study 1a, where the encoding context presented *no* alliance information. The drop in effect size for race, from r = .74 to r = .54, makes sense if the alliance detection system had encoded the men's charity group membership and registered that it may be a relevant dimension of alliance, independent of and uncorrelated with race. It also makes sense that this decrease would be more modest and fragile than the decreases found in the coalitions-relevant conditions, where the recall context suggests that charity group membership is an alliance category of immediate relevance to the targets.

#### Are visual markers of coalition membership necessary to reduce categorization by race

No. In Study 3, membership in *Habitat* versus *Partners* had to be inferred on the basis of conversational cues alone; there were no visual markers of charity group membership because everyone was dressed in gray shirts. Compared to the gray shirt conditions of Study 1, where coalitions were absent, racial categorization was reduced by the introduction of cross-cutting coalitions—dramatically so when the recall context suggested that these coalitional groupings were important (i.e., in the coalitions-relevant conditions). Indeed, race was “erased” for female targets in these conditions—it was not retrieved to a significant extent.

## When coalitions are turned into visually-distinct social categories, what happens to categorization by coalition, race, and sex? Studies 5 and 6 (Detailed methods and results in File S1)

By hypothesis, the alliance detection system detects and encodes perceptual cues that predict alliances, such as manner of dress, speech, family resemblance, and so on. This design feature allows possible allies to be identified at a later time, before they are engaged in a cooperative project or conflict. In this view, a difference in shirt color that predicts alliances will be stored as an alliance cue, which can be used at retrieval.

**Figure 5:**
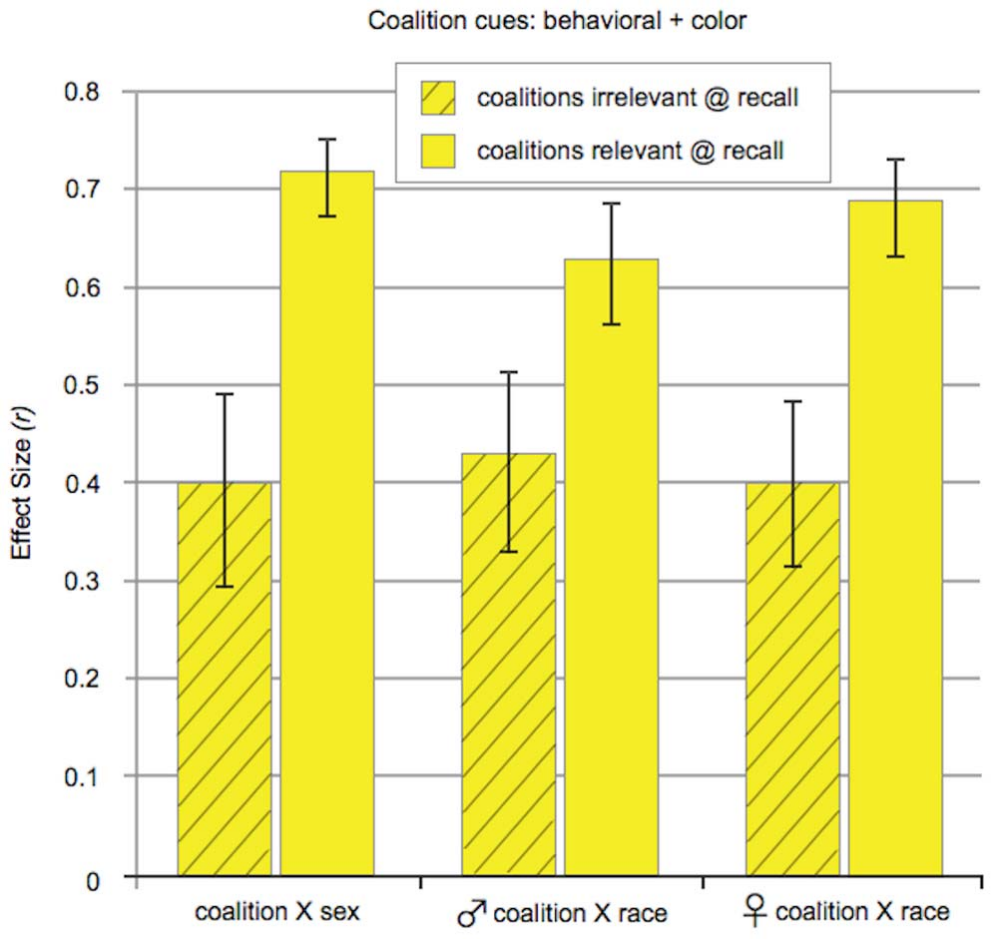
Effect sizes for categorization by coalition when membership is marked by visual and behavioral cues (Studies 5 and 6). Targets in these conditions wore different colored shirts, such that cues to coalition membership were both behavioral and visual. ♂, ♀ denotes sex of target stimuli. Coalitional categorization was stronger than in the gray conditions (Studies 3 and 4), but followed the same pattern. Error bars: +/− 1 S.E.

**Figure 6:**
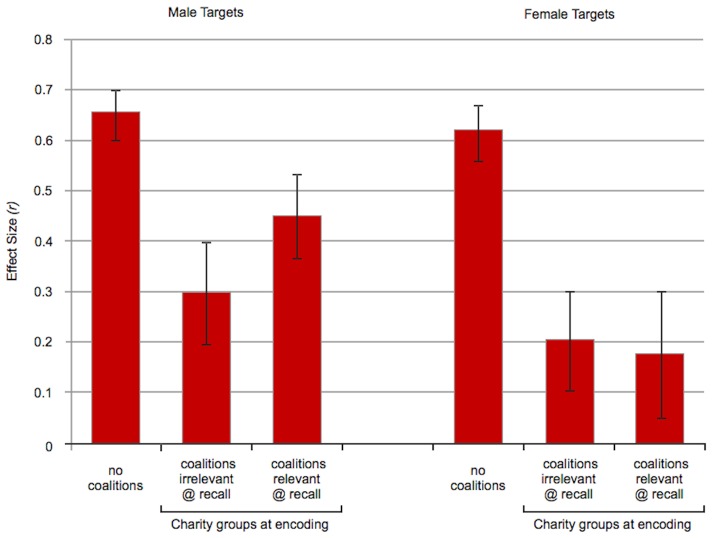
Racial categorization when coalition membership is marked by visual and behavioral cues (Studies 1 and 5). Race is crossed with shirt color in all of these conditions. Effect sizes for racial categorization are shown for male targets (left panel) and female targets (right panel). In each panel, the leftmost bar (labeled “no coalitions”) depicts racial categorization when coalitions were absent (Study 1)). The two bars to its right depict racial categorization when coalitions were present and marked by differences in shirt color (Study 5). Behavioral cues and visual cues to coalition membership were present at encoding for all the coalition conditions of Study 5. The sentences at recall varied across conditions: Their content was either relevant to coalition membership (“coalitions relevant @ recall”) or applicable to members of both coalitions (“coalitions irrelevant @ recall”). When each charity group is wearing different colored shirts, the recall context always has a least one cue that coalition membership is relevant to the targets (a visual cue). This single cue was enough to down-regulate racial categorization; there was no added benefit to having coalition-relevant sentences at recall. Racial categorization was as low in these conditions as in the gray ones with coalition-relevant sentences (Study 3). For female targets, race was “erased” in the color + coalitions-relevant condition, and almost so in the color + coalitions-irrelevant condition. Error bars: +/− 1 S.E.

**Figure 7:**
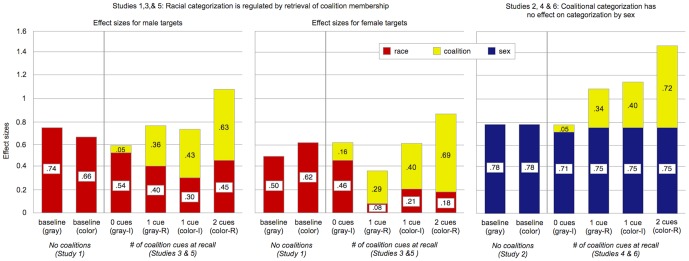
Categorization by coalition, race, and sex across six studies. Effect sizes for coalition (yellow bars) follow the same pattern across conditions. R and I refer to conditions in which the sentences at recall were coalition-relevant vs. coalition-irrelevant. When the recall context contains at least one cue that coalition-membership is relevant—either verbal (gray-R) or visual (color-I), racial categorization is down-regulated as much as when there are two cues (color-R). Gender categorization is not affected at all by coalitional manipulations. The different heights of the stacked bars show that categorization is not zero-sum: Retrieval of one category can be up-regulated without causing any decrease in retrieval of the crossed category.

Studies 5a, 5b, and 6 were identical to Studies 3a, 3b, and 4, except that members of *Habitat for Humanity* were wearing red shirts and members of *Partners in Health* were wearing yellow ones (instead of everyone wearing identical gray shirts). This change turns coalitions into visually-distinct social categories, recognizable from the photos. The main results are displayed in Figures 3 and 5–7. (Baseline measures of racial and gender categorization are from the color conditions of Studies 1 and 2, where coalitions were absent.)

Alliance variables regulated the retrieval of charity group membership in the same pattern as in the gray-shirt conditions (Studies 3a, 3b, 4; see Figure 5 and Figure S1 in File S1). The primary difference was that coalitional categorization was boosted across conditions when behavioral alliance cues were correlated with differences in shirt color—robustly so, unlike shirt color categorization when coalitions are absent (see Studies 1, 2 and SOM). When charity group membership was marked by two cues at recall—shirt color and coalition-relevant sentences—coalitional categorization was strongest (*r*s  = .63, .69, .72 respectively in Studies 5a, 5b, and 6). Caveats about structural fit inferences need to be considered, of course, when interpreting the magnitude of coalition effects in these particular conditions (see SOM for discussion). But categorization by charity group was enhanced even when the sentences at recall were coalition-*irrelevant*—conditions in which structural fit inferences could play no role.

Importantly, racial categorization was down-regulated whenever charity group membership was retrieved in these color conditions—even when the sentences at recall were coalition-irrelevant (see Figure 6). In contrast, coalitional variables had no effect on gender categorization, even though group membership was flagged by shirt color differences (see Figure 3). Categorization by sex was very strong when coalitions were absent (r = .78) and when coalitions were present (rs  = .75); this was true whether coalitional categorization was moderate (r = .40, coalitions-irrelevant condition) or very strong (r = .72, coalitions-relevant condition).

Why did coalitional categorization increase when charity groups were made visually-distinct? And why did this reduce racial categorization so deeply, even when the sentences at recall were coalition-irrelevant? During the encoding phase, it may be easier to track who is allied with whom when conversational alliance cues are flagged by color cues. A second possibility is that a recall context in which the targets are wearing coalitional colors suggests that coalition membership matters to them.

Outside the laboratory, people often signal their coalitional affiliations by wearing t-shirts with distinctive colors or slogans; the immediate context suggests whether those affiliations are currently relevant (e.g., four men wearing identical soccer jerseys suggests team-relevance if they are boarding a plane together, but not if they are scattered throughout the airport). A recall context in which the conversants are distinguishing themselves by wearing the colors of *Habitat* versus *Partners* suggests that differences in coalition membership remain relevant to them—it is a cue that charity group is a helpful alliance category to retrieve in this situation.

If this interpretation is correct, then priming an alliance category at recall should influence its retrieval, whether the prime is visual (race, shirt color) or verbal (coalition-relevant sentences). This pattern is clear in Figure 7, which shows categorization by coalition, race, and sex in every condition of Studies 1–6. Charity was not retrieved at all when the recall context had no cues that it was relevant; race was the only alliance category being primed at recall in the gray, coalition-irrelevant conditions. When a single cue primed the relevance of charity group at recall—either coalitional colors *or* coalition-relevant sentences—there was an up-regulation of coalitional categorization and a down-regulation of racial categorization, with similar magnitudes. Racial categorization was low whether these relevance cues were alone or combined: ∼.35 for male targets and ∼.15 for female targets. Yet gender categorization remained invariant, no matter how strongly we primed the relevance of charity group membership at recall.

### All else equal, is racial categorization stronger for male than for female targets?

Yes. A linear regression across all conditions in which coalitions were present (Studies 3 and 5) showed that race was categorized more strongly for male than for female targets (combined *M*_diff_ (SD): 1.28 (2.72) vs. 0.71 (2.78), *t*(572)  = 2.56, *p* = .011, *r* = .10). The difference was largest in the coalition-relevant conditions, which elicited virtually no categorization by race for female targets (male vs. female targets: *t*(262)  = 2.69, *p* = .008, *r* = .16). It was easier to “erase race” in female than male targets—especially when the recall context suggested a situation in which alternative (non-racial) alliance categories are relevant. This is consistent with Sidanius and Pratto's hypothesis that categorization by race (a culturally-specific alliance category) should be especially activated by male coalitions [2].

## General Discussion and Conclusions

Small-scale warfare between coalitions was common among ancestral hunter-gatherers [33], [34] and remains a signal characteristic of chimpanzee societies [50], [51]. Given this long evolutionary history, it is not surprising that coalitional aggression—cooperation in the service of conflict—has inspired many models of the evolution of group cooperation and the adaptations that support it [2], [8], [52], [53].

War and conflict are not, however, the only situations in which it is important to know who is allied with whom, ancestrally or now. Our foraging ancestors formed cooperative alliances to achieve many common goals, from procuring resources to providing assistance. Detecting alliances is important for understanding and participating in these peaceful coalitions, as well as competitive ones. An alliance detection system—if it exists—should be activated by situations indicating that several individuals are disposed to cooperate with one another. Competition, conflict, or antagonism might help, but they should not be necessary.

### Testing for design features

To test this prediction about the system's scope, we created a peaceful scenario in which members of two coalitions were having a friendly conversation. The coalitions were charity groups, each composed of individuals who cooperate with one another to achieve a common goal: helping others. During the conversation, these charitable individuals contributed information about how they prepare for and carry out their cooperative activities. Some of these comments were coalition-specific; others were very general. To isolate the effect of behavioral cues to cooperation, there were no visual distinctions marking off coalition membership in the main studies (3, 4); members of both charity groups were wearing gray shirts.

#### Cooperation—even when peaceful—regulates the encoding and retrieval of alliance categories

Participants responded to conversational cues of cooperation by implicitly assigning the speakers to charity-based alliance categories. These alliance categories were retrieved whenever the social context at recall implied they were relevant to the speakers.

There was coalitional categorization even when the relevance cues at recall were purely conversational—comments about activities that differentiate the two charity groups, made by identically-dressed speakers (Studies 3, 4). Similar levels of coalitional categorization were found when the relevance cues at recall were purely visual—speakers wearing different coalitional colors while making general comments (Studies 5, 6.). Charity group was retrieved in the same pattern, whether it was crossed with race or sex (see Figure 7, yellow bars).

Many obvious distinctions—even visual ones—do not elicit categorization in WSW studies (Studies 1, 2). Yet people spontaneously categorized individuals by their alliances based on implicit conversational cues, both here (Studies 3, 4) and in Kurzban et al [1]. This suggests that an alliance detection system exists, and peaceful cooperation falls within its scope.

#### Coalitional cooperation can reduce categorization by race

As a strong test of the alliance system's retrieval functions, we crossed charity group membership with race (Studies 3, 5)—a visually-distinct social category that is difficult to suppress [14], [16], [19], [22]. If the color of our skin is encoded and retrieved because perceptual systems cannot do otherwise, then racial categorization should be impervious to alliance-relevant variables. But if race is spontaneously retrieved because differences in skin color have acquired predictive validity as alliance cues in our social ecology, then the visual salience of race should be easy to defeat. Behavioral evidence that cooperative alliances are currently in play, but that race does not predict who is allied with whom, should diminish the retrieval of racial categories.

By comparing levels of racial categorization when coalitions were present versus absent, we were able to detect what conditions reduce the retrieval of race without relying on visual differences between coalitions (Study 1 vs. 3). Behavioral evidence for cooperative affiliations was sufficient to reduce the retrieval of race; there was no need to introduce features that visually group the targets by coalition. When comments at recall suggested that differences in coalition membership were relevant to the speakers—all of whom were wearing identical gray shirts—racial categorization was cut almost in half for male targets, and was eliminated for female targets. The reduction in racial categorization in these behavior-only conditions was as large or larger than the reductions found when the targets were wearing different coalitional colors at recall (Study 1 vs. 5).

#### Alliance categories are encoded promiscuously, but retrieved selectively

A nimble alliance retrieval system—one that dynamically updates the values it assigns to candidate alliance cues (including race)—should respond most powerfully to signs of cooperative affiliations when they appear at recall: the situation happening *now*. For a sensitive test of this hypothesis, we provided coalitional information during the encoding phase of the charity studies, but varied the social context at recall.

The most revealing cases are those in which both charity groups are wearing identical gray shirts; this makes race the only visually-distinct social category, and behavior the only basis for tracking alliances. When the comments appearing at recall suggested that differences in charity group were relevant to the speakers, the retrieval of charity-based alliance categories was up-regulated, and the retrieval of racial categories was strongly down-regulated. Results were different when the recall context lacked cues of coalition relevance—that is, when identically dressed targets were making comments about common, rather than coalition-specific, activities. Charity group membership was not retrieved at all in these conditions, and race was down-regulated modestly (male targets) or not at all (female targets).

Many WSW studies refer to social “categorization”, without distinguishing category encoding from category retrieval. The experimental design we used dissociated the two processes. The results indicate that newly introduced alliance categories are encoded and stored as the information becomes available, but retrieved selectively in response to cues that they are relevant to the interaction in progress. They also show that race behaves like an alliance category: The same coalition-relevant variables that up-regulated the retrieval of charity-based alliance categories also down-regulated the retrieval of racial categories. But they had no effect on the retrieval of gender categories (as predicted).

### Eliminating alternative explanations for the reduction in racial categorization

#### General priming

The fact that categorization by race (and charity group) were sensitively regulated by coalitional variables cannot be dismissed as a general “priming effect”. General relevance primes—ones that are alliance-neutral—have no effect on racial categorization (e.g., targets conversing about how to improve British education (race-neutral) vs. race-relations (race-relevant) [16]; or, when race and sex are crossed, telling participants that sex [vs. race] can influence people's judgments, so “pay close attention to the speakers' sex [vs. race]” [19]). In contrast, racial categorization did respond to cues that an alternative alliance category was relevant to the speakers.

The speed and specificity of this effect was remarkable. The conversation presenting information about the two charity groups was very recent (it ended only one minute before the recall phase). In spite of this, there were large differences in how strongly race (and coalition) were retrieved, which reflected a subtle difference in the set of comments presented at recall. When these comments referred to *differences* in what the two coalitions do, there were deep reductions in racial categorization. When they referred to more general charitable activities—“priming” charities, but not differences in dispositions to cooperate—the reductions in race were either modest (male targets) or non-existent (female targets). This applied only to race; both sets of comments supported strong categorization by sex.

That this subtle difference in coalition-relevance matters for racial categorization, while major differences in race-relevance do not, is telling. It implies the existence of a retrieval system that is specialized for tracking alliances—one that monitors ongoing events for fresh clues about dispositions to cooperate, and uses them to rapidly update cue validities for candidate alliance categories, including race.

#### Is crossing categories sufficient

Theories invoking similarity-based or perceptual categorization suggest a counter-hypothesis: that crossing race with a second category changes perceptions of similarity, and this is sufficient to decrease categorization by race [30], [31]. In this view, there is nothing special about coalitions; the same effect can be achieved by crossing race with a strong visual difference having nothing to do with alliances.

The results clearly rule out this counter-hypothesis: When coalitions were absent, crossing race with a striking color difference—red versus yellow shirts—had no effect on racial categorization (Study 1). Gender categorization did not change either, whether it was crossed with shirt color (Study 2) or coalitions (Study 4) or both (Study 6). Therefore, the introduction of a cross-cutting category cannot, by itself, explain the effect of coalitional variables on racial categorization.

#### Competitive category retrieval

Racial categorization decreased when coalitional categories were retrieved. Was this because domain-general retrieval processes were competing for some common cognitive resource? The studies crossing charity group with sex eliminate most (perhaps all) alternative explanations of this kind (Study 2, 4, 6). Like race, sex is a visually-distinct social category. Like race, sex elicits social inferences and responses. Yet the same alliance variables that so precisely regulated categorization by race had no effect on categorization by sex. Sex was retrieved at very high levels whether coalitions were present or absent, relevant or irrelevant, and regardless of how strongly coalition membership was retrieved. This is clear from Figure 7—gender categorization did not decrease even when coalitional categorization was very strong. This means that the effects of coalitional variables on race cannot be explained by limits on attention, working memory, or any other factor that might produce competitive category retrieval.

### Selective effects: Race responds like an alliance category, sex does not

A system that is well-engineered for tracking cooperative alliances should have retrieval functions that discriminate between race and sex—that is, between alliance categories and other categories. Although any cue, including sex, can become an alliance cue given the right situation, gender categories did not arise because they predict cooperative alliances. They are so fundamental to mammalian social life that they are probably constructed by mechanisms specialized for that function, and they should be reliably retrieved by a large number of motivational systems.

The genesis of racial categories should be different. Racial categories vary widely across cultures and time [54] because they reflect patterns of cooperation, conflict, and social division that are semi-stable, but eventually change [2], [55]. We propose that an alliance system registers these patterns, creating categories such as *black* and *white.* In this view, racial categories are creatures of the alliance system, which retrieves them most strongly when the situation suggests they are the best (or only) cues available for predicting who is allied with whom. Racial categorization seemed difficult to suppress in prior research because prior efforts did not manipulate coalitional variables. In support of this claim, we have shown that it is easy to down-regulate racial categorization by manipulating alliance variables.

As predicted, these effects were selective: The same coalitional variables that so sensitively regulated categorization by race—a putative alliance category—had no effect at all on categorization by sex. By manipulating alliance-relevant variables, we produced a functional dissociation between racial categorization and gender categorization—further evidence that coalitional variables engage a cognitive system specialized for tracking alliances.

### An adaptive specialization?

The alliance detection hypothesis survived multiple tests of its core predictions, using a design that eliminates alternative explanations and isolates the effects of cooperation. Cues of coalitional cooperation had specific, selective, sensitive effects on the encoding and retrieval of charity group and race, but not sex. Categorization systems with a more general function would not produce this particular pattern. Taken together, the results implicate a cognitive system specialized for constructing and regulating the retrieval of alliance categories.

### Does coalitional conflict matter at all?

It is too early to tell. When the faces (and participant population) were the same, effect sizes for coalitional and racial categorization in the charity studies were similar to those for the fighting basketball teams tested by Kurzban et al. [1]. On the other hand, sex was categorized at even higher levels for the fighting teams than for the peaceful charity groups, and declined slightly when team was marked by shirt color. This could, of course, reflect random variation. But it is also possible that coalitional conflict affects the retrieval of sex differently than peaceful cooperation; this would be reasonable given that gender powerfully organizes warfare among human foragers and chimpanzees [2], [51].

Consistent with social dominance theory [2], coalitional variables that eliminated racial categorization for female targets merely decreased racial categorization for male targets, supporting the view that the alliance system's design was shaped, in part, by selection pressures for male coalitional aggression [2], [53]. If so, then the system may be designed to use sex of target in revising its estimate that race (or any other axis of alliance) has become irrelevant. In this view, situational cues would down-regulate—but not eliminate—the retrieval of any pre-existing alliance category that has a high prior probability of organizing the alliances of men.

There is something salutary about the fact that racial categorization can be so easily diminished, without the need for common enemies and group antagonism. Cooperation trumps the color of our skin. Exposing people to a brief, peaceful interaction involving two groups of coalitional cooperators, for whom race did not predict group membership, was sufficient to reduce, and sometimes eliminate, the retrieval of their race. It raises the possibility that, as cooperation across racial lines continues to increase in our society, race too will fade in relevance.

## Supporting Information

File S1 **This file contains Figure S1 and Tables S1–S4.** Figure S1, Coalitional categorization; charity groups present at encoding. Table S1, Racial categorization with coalitions absent (Study 1) vs. present (Study 3). Table S2, Coalitional categorization when crossed with Race (Studies 3 & 5) or Sex (Studies 4 & 6) and shirt color categorization when crossed with Race (Study 1) or Sex (Study 2), Table S3, Shirt color categorization without coalitions. Sex of participant effects, Study 1. Table S4, Gender categorization with Coalitions Absent vs. Present (Study 2 vs. Study 4, 6).
(PDF)
