# Supplementary material for: The Content of Our Cooperation, Not the Color of Our Skin: An Alliance Detection System Regulates Categorization by Coalition and Race, but Not Sex
Source: PLoS One. 2014 Feb 10;9(2):e88534. doi: 10.1371/journal.pone.0088534 (PMC3919763; doi:10.1371/journal.pone.0088534)
Supplement: File S1 — This file contains Figure S1 and Tables S1–S4. Figure S1, Coalitional categorization; charity groups present at encoding. Table S1, Racial categorization with coalitions absent (Study 1) vs. present (Study 3). Table S2, Coalitional categorization when crossed with Race (Studies 3 & 5) or Sex (Studies 4 & 6) and shirt color categorization when crossed with Race (Study 1) or Sex (Study 2), Table S3, Shirt color categorization without coalitions. Sex of participant effects, Study 1. Table S4, Gender categorization with Coalitions Absent vs. Present (Study 2 vs. Study 4, 6). (PDF) [file pone.0088534.s001.pdf]

Supplementary Online Material

Contents:

- ***Studies 5 & 6.*** When coalitions are present and visually-distinct: Methods and results
- ***Table S-1.*** Racial categorization with coalitions absent (Study 1) vs. present (Study 3)
- ***Table S-2.*** Coalitional categorization when crossed with Race (Studies 3 & 5) or Sex (Studies 4 & 6) and shirt color categorization when crossed with Race (Study 1) or Sex (Study 2)
- ***Table S-3.*** Shirt color categorization without coalitions. Sex of participant effects, Study 1
- ***Table S-4.*** Gender categorization with Coalitions Absent vs. Present (Study 2 vs. Study 4, 6)
- ***Sentences used as stimuli.*** Coalitions present conditions, Coalitions absent conditions

Studies 5 & 6: When coalitions are present and visually-distinct: Crossing coalition membership with Race (Study 5) and Sex (Study 6)

Because behaviors that reveal alliances are transitory, Kurzban et al [1] predicted that perceptual cues that predict alliances but are more stably present should be detected by the alliance system, and encoded as alliance cues. This could include manner of dress, gait, speech, family resemblance, and so on. This design feature allows potential allies to be identified in advance of organizing a group project or recruiting support in response to a threat. In this view, a difference in shirt color that predicts alliances will be stored as an alliance cue, and used at retrieval.

In Studies 5 and 6, behavioral cues to coalition membership were supplemented by the addition of colorful visual markers: targets from one coalition wore red shirts and targets from

the other coalition wore yellow shirts. Thus shirt color was a visually salient cue to coalition membership, and this visual cue—like race and gender—was present at both encoding and recall.

These *color conditions* were designed to be focused contrasts to the color conditions of Studies 1 (race) and 2 (sex), in which there was no coalitional information. We also compare them to the otherwise identical gray conditions of Studies 3 and 4, in which the only cues to coalition membership were behavioral. This allows us to see what happens to categorization by coalition, race, and sex when coalition membership is made into a visually distinct social category, like race and sex.

Method

Ethics Statement

All of the research reported herein was approved by the Human Subjects Committee at the University of California, Santa Barbara. Written informed consent was obtained from all participants.

Participants

Study 5 had 290 participants (159 female, 1 unknown), mean age 19.76 (SD 3.23). Study 6 had 138 participants (74 female), mean age 19.54 (SD 1.66).

Design

Visually distinct coalitions were crossed with race in Study 5. Its design was identical to the design of Study 3 (5a: male targets; 5b: female targets). Study 6 crossed visually distinct coalitions with sex. Its design was identical to that for Study 4.

Materials and Procedure

Study 5: Coalition x Race. The procedure for Study 5 was identical to that for Study 3. The target photos were also identical except for one thing: In the color conditions of Study 5, members of *Habitat for Humanity* wore red shirts and members of *Partners in Health* wore yellow shirts.

Because the conversation always alternated back and forth between the two coalitions, shirt color also alternated back and forth in Study 5—red, yellow, red, yellow—just as it did in Study 1, in which coalitions were absent.

Study 6: Coalition x Sex. The procedure for Study 6 was identical that for Study 5. Only the photos were different: The targets differed in sex rather than race. The photos were the white male and female targets from Study 5 (and identical to the color conditions of Study 2, in which coalitions were absent).

Measuring coalitional categorization: structural fit versus retrieval context

One goal of Studies 5 and 6 is to measure coalitional categorization when groups are interacting peacefully, while keeping the method as parallel as possible to the high conflict scenarios used by Kurzban et al. [1]. This requires a condition in which each coalition is wearing a different color, and the sentences at recall are coalition-relevant. This presents a problem that did not arise in the color conditions of the Kurzban et al. study.[1]

Consider the coalition-relevant conditions of Study 5, in which each charity wears a different color. As the conversation unfolds, participants may notice that volunteers from

[1] Kurzban et al. [1] was designed to eliminate (or at least minimize) inferences due to structural fit. The sentences presented at recall were coalition-relevant: they expressed antagonism between the two teams, implying that team membership was a relevant alliance category to retrieve. But they were not coalition-*specific*: each sentence said by one team was followed by a very similar sentence said by the other. Coalition membership was implicit in the order in which the angry sentences appeared during the initial, encoding phase. At recall, these pragmatic cues were no longer present, because the sentences were presented in random order. Because the semantic content of the sentences was so similar across the two teams, it would be difficult to infer the speaker's team membership from a sentence that had been removed from its pragmatic context at encoding.

Partners wear yellow and Habitat wear red. If they do, they could generate a same-coalition error by clicking on a target with the right color shirt in response to a coalition-relevant sentence, *even if they remember nothing about the sentence or the speaker*. This is because (i) the coalition-relevant sentences have content that allows one to infer which category the speaker belongs to (Habitat or Partners), and (ii) this inference can be matched to a visual cue of category membership (shirt color). When both conditions jointly hold, there is a "structural fit" between sentences and photos [2]. Structural fit complicates the interpretation of categorization scores because it allows same-category errors to arise through two different cognitive processes.

For example, "Some of us are nutritionists, some trained in agriculture" implies that the speaker belongs to Partners in Health and, therefore, wore yellow. When mistakes are made, all misattributions to yellow-shirted targets will be coded as same-coalition errors. Many of these will arise because participants encoded and subsequently retrieved representations that include the coalition membership of the speaker and other targets—which is what the memory confusion paradigm is intended to measure. But some may occur by inference alone. These same-coalition errors will increase the categorization score, without reflecting the extent to which participants encoded and retrieved the charity group membership of individual targets. For this reason, it is possible to *over*estimate coalitional categorization when the sentences at recall are coalition-specific (as they are in the coalition-relevant conditions) *and* charity group membership is marked by color [3,4]. (Structural fit cannot inflate measures of categorization by race (or sex) in any condition, because there are no sentences that allow one to infer the speaker's race (or sex). Additionally, assignment of targets to sentences is random with respect to race and sex for 20 of the sentences.)

Note that structural fit does not arise in the coalitions-relevant conditions of Studies 3 or 4 (gray conditions). At recall, it was possible to infer the speaker's coalition membership from the content of the sentence presented. But when everyone is dressed in gray, there is no visual cue of group membership to which such inferences can be matched. Stored representations of the targets that pair their faces with their coalition membership must be retrieved to generate more same-coalition than different-coalition errors than expected by chance. This is true whether the participant has stored the speaker's charity group membership with a previously encoded representation of the coalition-relevant sentence, or inferred it from that sentence when it was presented at recall.

Using coalition-irrelevant sentences at recall in the color conditions provides one solution. Because the content of these sentences does not allow participants to infer the speaker's coalition membership, there is no possibility of overestimating coalitional categorization due to structural fit. Yet the results of Studies 3 and 4 show that recall context matters: The use of coalition-irrelevant sentences at recall is likely to *under*estimate the extent to which coalitional information was encoded and stored.

Bracketing coalitional categorization. When charity group membership is marked by shirt color, as in Studies 5 and 6, there is no unbiased method of measuring coalitional categorization using a standard memory confusion paradigm. *Coalitions-irrelevant* conditions may underestimate coalitional encoding and storage because the sentences presented at recall suggest a situation in which engaging in charitable activities is relevant, but distinguishing speakers by their group membership is not. *Coalitions-relevant* conditions may overestimate coalitional encoding and retrieval due to structural fit inferences. We can bracket the coalition

effect size, however, by comparing coalition-relevant with coalition-irrelevant conditions in these color conditions.

Results and Discussion

Coalition results (Studies 5 and 6: color conditions; see *Table S-2*)

How strongly is coalition membership categorized when behavioral cues are reinforced by a colorful visual marker?

Coalitions-relevant condition. When the sentences presented at recall were coalition-relevant, participants made more same-coalition errors than different-coalition errors whether coalition was crossed with race (5a, male targets: $p = 10^{-8}$, *df* 72; 5b, female targets: $p = 10^{-9}$, *df* 60) or sex (6: $p = 10^{-8}$, *df* 68). The effect sizes were large and similar across studies: $r = .63$, .69, and .72, respectively, for Studies 5a, 5b, and 6.

Coalitions-irrelevant condition. Even though the sentences presented at recall could have been said by members of either charity group, when the color cue to coalition was present, participants made more same-coalition errors than different-coalition errors. This was true whether coalition was crossed with race (5a, male targets: $p = .0002$, *df* 70; 5b, female targets: $p = .0001$, *df* 84) or sex (6: $p = .0007$, *df* 68). The size of the coalition effect was almost identical across the three studies: $r = .43$, .40, and .40, respectively for Studies 5a, 5b, and 6.

Bracketing the effect size for coalitional categorization. These two conditions bracket the effect size for categorization by charity group when colorful markers are flagging membership; it can be estimated as $r = .40$-.72. The lower values, from the coalition-irrelevant conditions, are free from structural fit, but may under-estimate encoding and storage for the reasons explained in

the main text—the sentences suggest that charitable activities, not coalition membership, are relevant to the targets. (*N.B.*: The difference in shirt color may suggest membership is relevant to them nevertheless). But are the higher values, from the coalition-relevant conditions, overestimates due to structural fit? These are the only conditions in which structural fit could have added to effect sizes.

Because the alliance detection system is designed to infer alliances and pick up predictive cues, inferences generated by this system may have contributed to these scores. However, the data suggest that strategic guessing—deliberative inferential strategies—played little if any role. Participants had as much time as they wanted to respond and the sentence content permits strategic inferences, yet the effect sizes for coalitional categorization in these conditions (r = .63, .69, .72) are the same or lower than many found in Studies 1-4 for race and sex (where structural fit can play no role; see Figures 4, 5, 7). They are also lower than those found for sex by Kurzban et al—r = .91, .84—which demonstrate that the memory confusion paradigm can elicit effect sizes close to r = 1.0 based on memory representations alone. Bypassing memory by using a deliberative guessing strategy could have produced nothing but same-coalition errors, with sky-high effect sizes. The effect sizes for Studies 5 and 6—which are high but not unusually so—are more consistent with the hypothesis that participants were simply following the instructions, which were to *recall* who said what.

Is coalition membership retrieved more strongly when the sentences at recall imply it is relevant than when they do not?

Yes. The effect sizes were always larger when the sentences at recall implied group membership was relevant than when they did not—.63 vs. .43, .69 vs. .40, and .72 vs. .40,

respectively for Studies 5a, 5b, and 6. A linear regression in which study (5a, 5b, 6) and participant sex were entered as co-variates confirmed this, showing that coalitional categorization was stronger when the sentences at recall were coalition-relevant than when they were coalition-irrelevant ($F(1, 423) = 67.25$, $p = 10^{-14}$, $r = .37$); there were no effects due to study or participant sex, and no interactions. Separate linear regressions confirmed that the difference due to coalition-relevance was present in each study separately (5a, male targets: $t(140) = 3.69$, $p = .0003$, $r = .30$; 5b, female targets: $t(143) = 4.64$, $p = 10^{-5}$, $r = .36$; 6: ($t(135) = 5.68$, $p = 10^{-7}$, $r = .44$).

The effect of coalition-relevance cannot be due to differences in encoding because participants were exposed to exactly the same set of behavioral and visual coalition cues during the encoding phase of each study. This pattern is what one would expect, however, if the alliance system is designed to encode alliances promiscuously but retrieve them selectively. Shirt color is the only cue that membership in different coalitions might matter when the sentences at recall could have been said by members of either charity group. But when the recall context joins differences in shirt color to sentences that mention activities that differ by coalition, there are two cues that remembering which charity group a speaker belongs might be important.

Structural fit inferences in the *coalitions-relevant+color* condition may have inflated the difference in retrieval of coalition membership in these color conditions, of course. By comparing coalitional categorization in the color conditions to the gray ones (where structural fit does not exist), we can get a sense of how much structural fit influenced these measures.

Was there an increase in coalitional categorization when behavioral alliance cues were reinforced by the addition of a colorful marker?

Yes. This can be seen by comparing coalitional categorization in the gray conditions of Studies 3 and 4 to the color conditions of Studies 5 and 6, holding all else constant.

Coalition-irrelevant conditions. When the sentences at recall were coalition-irrelevant—eliminating any possibility of overestimating due to structural fit—coalitional categorization was stronger when colorful markers reinforced behavioral cues at encoding (and were present at recall) than when these visual markers were absent (3a vs. 5a, male targets: $t(142) = 2.63$, $p = .01$, $r = .21$; 3b vs. 5b, female targets: $t(163) = 2.12$, $p = .035$, $r = .16$; 4 vs. 6: ($t(137) = 2.23$, $p = .027$, $r = .19$)). Descriptively, this corresponded to effect sizes of $r = .05$ (gray) vs. $r = .43$ (color) for male targets differing in race (Study 5a); $r = .16$ (gray) vs. $r = .40$ (color) for female targets differing in race (Study 5b); and $r = .05$ (gray) vs. $r = .40$ (color) for targets differing in sex (Study 6).

Coalition-relevant conditions. The same pattern was found when the sentences at recall were coalition-relevant, where structural fit inferences could have contributed to the difference between gray and color conditions (3a vs. 5a, male targets: $t(142) = 4.31$, $p = 10^{-4}$, $r = .34$; 3b vs. 5b, female targets: $t(117) = 4.72$, $p = 10^{-5}$, $r = .40$; 4 vs. 6: ($t(133) = 5.55$, $p = 10^{-7}$, $r = .43$). Descriptively, this corresponded to effect sizes of $r = .36$ (gray) vs. $r = .63$ (color) for male targets differing in race; $r = .29$ (gray) vs. $r = .69$ (color) for female targets differing in race; and $r = .34$ (gray) vs. $r = .72$ (color) for targets differing in sex.

Overall results. For coalition-relevant conditions, the effect sizes associated with the difference between color and gray conditions were a bit larger (.34, .40, .43) than those for the coalition-irrelevant conditions, where structural fit could play no role (.21, .16, .19). To get overall measures, we conducted a two-way ANOVA controlling for study (*n.s.*, $p = .28$) and

participant sex (*n.s., p* = .77). This analysis revealed a main effect of marking coalition with shirt color (gray vs. color: $F(1, 845) = 90.15$, $p = 10^{-19}$, $r = .31$), a main effect of having coalition-relevant sentences at recall (coalition-relevant vs. irrelevant: $F(1, 845) = 70.17$, $p = 10^{-15}$, r = . 28), and an interaction between them ($F(1, 845) = 24.52$, $p = 10^{-6}$, $r = .17$).

The significant interaction reflects a greater degree of coalitional categorization when both coalitional cues were present at recall: that is, in the color + coalitions-relevant conditions. These are the only conditions in which scores for coalitional categorization could have been enhanced by structural fit inferences. They are also the only conditions in which there are two sets of cues at recall suggesting that coalition membership is relevant to the targets (visual and verbal). It is difficult to say which is the more important factor, but as Figure S1 shows (and the analysis of coalition-irrelevant conditions demonstrates, above), the boost in coalitional categorization due adding color is a true main effect.

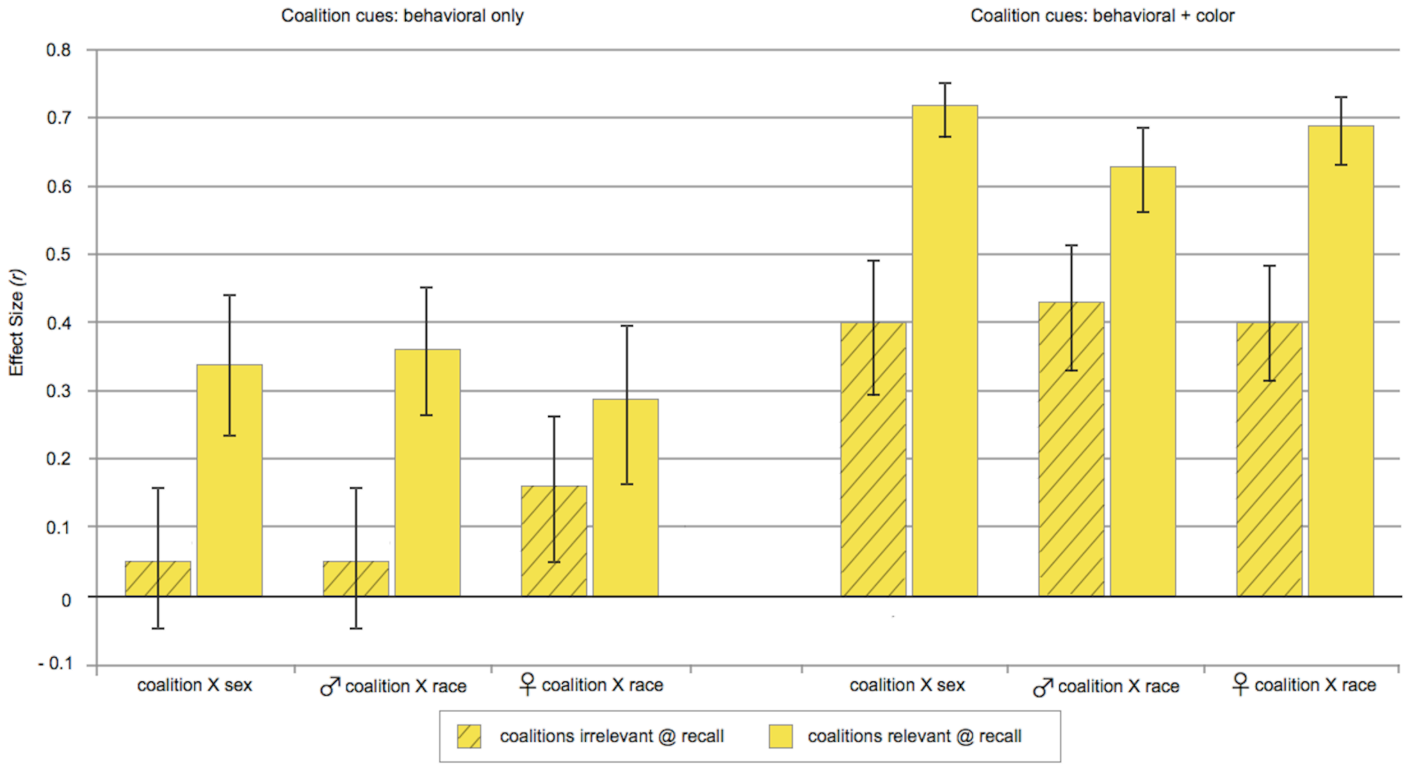

Supplementary Figure S1. Coalitional categorization; charity groups present at encoding.

Does categorization by shirt color vary depending on whether it marks coalitions?

Yes. This can be seen by comparing levels of categorization by shirt color in Studies 1 and 2, where the color difference had no social meaning, to levels in Studies 5 and 6, where it marked charity group membership.

Studies 6 and 2: Coalition x Sex. When shirt color had no social meaning (Study 2), participants did not categorize targets by their shirt color at all. Categorization by shirt color in Study 6, where it marked coalition membership, was stronger than in Study 2, where it did not. This was true whether the sentences at recall were coalition-irrelevant ($t(134) = 2.61$, $p = .01$, $r = .22$) or coalition-relevant ($t(134) = 7.57$, $p = 10^{-11}$, $r = .55$).

Studies 5 and 1: Coalition x Race. When shirt color had no social meaning (Study 1), participants did not categorize same sex targets by their shirt color at all ($r = .02, -.15$); only opposite sex targets were categorized by their shirt color ($r = .41, .33$). By contrast, subjects categorized the coalition/shirt color of same sex and opposite sex targets with equal strength when shirt color marked coalition membership (Study 5).[2] Unlike Study 1, however, there were no sex differences in Study 5. This means that shirt color is treated differently when it marks coalition membership than when it does not.

Coalitions-irrelevant conditions. For the coalitions-irrelevant conditions of Study 5, same-sex targets were categorized by shirt color (coalition) at higher levels than in Study 1, where shirt color had no meaning (men viewing male targets, $t(63) = 2.23$, $p = .03$, $r = .28$; women viewing female targets, $t(86) = 2.57$, $p = .012$, $r = .27$). Opposite-sex targets were

[2] For example, in the coalitions-irrelevant conditions, coalitional categorization was as follows. For male targets: male participant $M_{\text{diff}} = 1.65$ (SD 3.54), $p = .01$, $r = .43$, female participant $M_{\text{diff}} = 1.46$ (SD 3.04), $p = .006$, $r = .44$. For female targets: female participant $M_{\text{diff}} = 1.34$ (SD 3.46), $p = .007$, $r = .36$, male participant $M_{\text{diff}} = 1.67$ (SD 3.28), $p = .006$, $r = .46$.

categorized by shirt color at the same (moderate) levels in Study 5 as they were in Study 1 (women viewing male targets: $t(72) = 0.255$, $p = .80$, $r = .03$; men viewing female targets: $t(71) = 1.32$, $p = .16$, $r = .15$).

Coalitions-relevant conditions. For this recall context, the difference between Study 5 and Study 1 was especially large, corresponding to effect sizes of .63 vs. .27 for male targets, and .69 vs. .12 for female targets (linear regressions controlling for participant sex: male targets, $t(139) = 5.10$, $p = 10^{-6}$, $r = .40$; female targets, $t(134) = 6.96$, $p = 10^{-10}$, $r = .51$). Structural fit inferences may have contributed to these large differences, but they could have done so only if participants were using shirt color as a coalitional marker in Study 5.

Coalitional categorization with and without conflict.

In the color conditions of Studies 5 and 6, the two charitable coalitions were chatting amiably. The effect sizes for coalitional categorization were $r = .63, .69, .72$ respectively (5a, 5b, 6). These are almost as high as those found for the color conditions in Kurzban et al (2001) conflict studies using the same male faces: $r = .79$ for male coalitions in conflict and $r = .81$ for mixed sex coalitions.

Gender results (Study 6: color conditions; see *Table S-4* **)**

Is categorization by sex reduced when peaceful coalitions interact, and sex does not predict who is allied with whom?

No. In Study 2, when there were no cross-cutting coalitions but targets differed in shirt color (half wearing red, half wearing yellow) the effect size for categorization by sex was $r = .78$.

In Study 6, where cross-cutting coalitions were present and flagged by differences in shirt color, categorization by sex was the same.

Coalitions-relevant condition. Participants strongly categorized targets by their sex—$r = .75$—making more same-sex than different-sex errors ($p = 10^{-13}$, *df* 68). Linear regression showed that this gender categorization score was no different from that found in the color condition of Study 2, which had no coalitions ($t(134) = 0.32$, $p = .75$, $r = .03$) or in the matching gray condition of Study 6, which had cross-cutting coalitions ($t(133) = 0.08$, $p = .93$, $r = .01$).

Coalitions-irrelevant condition. Categorization by sex was just as strong in this condition —$r = .75$—with participants making more same-sex than different-sex errors ($p = 10^{-13}$, *df* 68). Gender categorization in this condition was no different from that found in the color condition of Study 2, which had no coalitions ($t(134) = 0.70$, $p = .48$, $r = .06$), in the coalitions-relevant condition of Study 6 ($t(135) = 0.36$, $p = .72$, $r = .03$), or in the matching gray condition of Study 6 ($t(133) = 0.43$, $p = .67$, $r = .04$).

Race results (Study 5: color conditions; see *Table S-1* **)**

Is categorization by race reduced when peaceful coalitions interact, and race does not predict who is allied with whom?

Yes. Study 1 measured how strongly participants categorized targets by their race when there were no cross-cutting coalitions. When half the targets were dressed in red and the other half in yellow, the effect size for categorization by race was $r = .66$ for male targets and $r = .62$ for female targets. In Study 5, where the same differences in shirt color marked cross-cutting coalitions, categorization by race was lowered. This was true whether the sentences at recall

were coalition-relevant or coalition-irrelevant: effect sizes for race were $r = .45$ and .30 for male targets, and $r = .18$ and .21 for female targets.

Coalitions-relevant condition. When the sentences presented at recall implied coalition membership was relevant, there were more same-race than different-race errors for male targets ($p = .0001$, *df* 72), but not for female targets ($p = .15$, *df* 60). Participants retrieved the race of male targets to a moderate extent ($r = .45$), but they did not retrieve the race of female targets to a significant extent ($r = .18$).

These racial categorization scores, which were found when coalitions were present and flagged by shirt color, are significantly lower than those found in Study 1, where coalitions were absent but targets differed in shirt color (linear regressions for male targets: $t(139) = 2.29$, $p = .023$, $r = .19$; for female targets: $t(134) = 2.91$, $p = .004$, $r = .24$).

Coalitions-irrelevant condition. The presence of cross-cutting coalitions led to a decrease in categorization by race when the sentences presented at recall were coalition-irrelevant, too. In these conditions, there were more same-race than different race errors for male targets ($p = .011$, *df* 70) and for female targets ($p = .057$, *df* 84). The effect sizes for race were $r = .30$ for male targets, and only $r = .21$ for female targets. These racial categorization scores are significantly lower than those found in Study 1, where coalitions were absent but the targets differed in shirt color (linear regressions for male targets: $t(136) = 3.51$, $p = .001$, $r = .29$; for female targets: $t(158) = 3.00$, $p = .003$, $r = .23$). In the color conditions of Study 5, there were no significant differences in racial categorization between the coalitions-irrelevant and coalitions-relevant conditions.

When coalition is marked by two cues at encoding—behavioral and color—is the decrease in racial categorization larger than when it is indicated by behavioral cues alone (Study 3 (gray conditions) vs. Study 5 (color conditions))?

Coalitions-relevant conditions. For this recall context, categorization by race was just as low in the gray conditions (behavioral only) as in the color conditions (behavioral + color marker): $r = .40$ vs. .45 for male targets ($t(142) = 0.61$, $p = .54$, $r = .05$), and $r = .08$ vs. .18 for female targets ($t(117) = 0.69$, $p = .49$, $r = .06$). (Moreover, all of these racial categorization scores were lower than the baselines established in Study 1).

Coalitions-irrelevant conditions. The color conditions elicited lower racial categorization than the gray ones only when the sentences at recall were coalition-irrelevant: $r = .54$ vs. .30 for male targets ($t(142) = 1.90$, $p = .059$, $r = .16$), and $r = .46$ vs. .21 for female targets ($t(162) = 2.03$, $p = .04$, $r = .16$).

This pattern implies that strong alliance cues at retrieval trump other factors. When every comment at recall referred to the different activities of *Habitat* versus *Partners*, this was sufficient to decrease racial categorization, virtually eliminating it for female targets. Nothing was added by including visual markers of coalition membership. When none of these comments referred to group membership, racial categorization decreased more when the recall context presented at least one cue that charity group membership was important: the different colored shirts of the targets.

Racial categorization with and without group conflict

In the charity studies reported here, racial categorization was reduced to about the same levels whenever the recall context presented at least one cue that membership in *Habitat* versus *Partners* was a relevant alliance category (Figure 8, main text). Providing two cues at recall—conversational *and* visual—was no more effective than a single conversational cue (gray conditions) or a single visual cue (coalitions-irrelevant conditions). Studies 1 and 5, in which all targets are wearing different color shirts, allow the most direct test of the efficacy of multiple alliance cues. The charity manipulation reduced racial categorization for male targets from a baseline level of $r = .66$ (without coalitions) to $r = .45$ (2 recall cues) and $r = .30$ (1 recall cue). The reduction in retrieval of race was even greater for female targets, from a baseline level $r = .62$ (without coalitions) to $r = .18$ (2 recall cues, *n.s.*) and $r = .21$ (1 recall cue). As in the gray conditions, when the recall context contained conversational alliance cues, the race of female targets was not retrieved to a significant extent.

This is different from the fighting, all-male basketball teams of Kurzban et al (2001), where two recall cues (verbal alliance cues + shirt color differences) elicited lower racial categorization than verbal alliance cues alone (gray conditions).[3] Further research could reveal whether this represents a difference in how the alliance system responds to coalitions in conflict compared with coalitions interacting peacefully. Group antagonism easily erupts into violence, where alliances can shift in highly volatile ways—particularly under ancestral conditions, where

[3] When the sentences at recall were irrelevant to coalition membership, Study 3 produced a similar pattern—lower racial categorization in color than gray conditions. In these conditions, the addition of shirt color as a coalitional marker created a difference in recall context (one membership-relevant alliance cue—shirt color— vs. none in the gray conditions). This interpretation is consistent with the pattern found across conditions in Studies 3 and 5. For example, racial categorization was also lower when there was one membership-relevant *conversational* cue compared to when there were no cues (both gray conditions). Note that in Studies 4 and 6, the same coalition-irrelevant sentences supported levels of gender categorization that were not only high, but equal in the gray and color conditions.

cross-cutting alliances based on kinship, marriage, and exchange created ties between individual members of rival bands and coalitions [4]. Consequently, the alliance detection system may be designed to respond to coalitional conflict by activating every alliance category suggested by the stimuli—which, in these experiments, includes race. If so, then suppressing the retrieval of race —or any other cross-cutting alliance category—may require a recall context with strong and redundant cues that it is irrelevant to the primary axis of conflict.

References

1. Kurzban R., Tooby J, Cosmides L (2001) Can race be erased? Coalitional computation and social categorization. Proc Natl Acad Sci U S A 98: 15387-15392.

2. Klauer KC, Wegner I (1998) Unraveling social categorization in the "who said what?" paradigm. J Pers Soc Psychol 75: 1155-1178.

3. Taylor SE, Fiske ST, Etcoff NL, Ruderman, AJ (1978) Categorical and contextual bases of person memory and stereotyping. J Pers Soc Psychol 36: 778-793.

4. Levine R, Campbell D (1972) Ethnocentrism: Theories of conflict, ethnic attitudes, and group behavior. New York: Wiley.

Table S-1. Racial categorization with coalitions absent (Study 1) vs. present (Studies 3 & 5)

	Errors: mean (standard deviation)						
	**same-race**	**different-race**	**difference scores**	***df***	***t***	***p***	***r***
**Gray conditions - Male targets**							
1a: No coalitions	6.44 (1.85)	3.58 (1.45)	2.87 (2.59)	70	9.33	10^{-13}	.74
3a: Coalitions Relevant	6.02 (1.78)	4.85 (1.39)	1.17 (2.68)	71	3.69	.0004	.40
3a: Coalitions Irrelevant	6.27 (1.71)	4.58 (1.39)	1.69 (2.67)	74	5.48	10^{-6}	.54
**Color conditions - Male targets**							
1a: No coalitions	6.49 (2.03)	3.98 (1.49)	2.51 (2.91)	65	7.19	10^{-9}	.66
5a: Coalitions Relevant	6.08 (1.97)	4.68 (1.31)	1.40 (2.79)	72	4.30	.0001	.45
5a: Coalitions Irrelevant	5.83 (1.71)	4.99 (1.46)	0.85 (2.72)	70	2.61	.011	.30
**Gray conditions - Female targets**							
1b: No coalitions	5.79 (2.03)	4.13 (1.53)	1.66 (2.89)	67	4.74	10^{-5}	.50
3b: Coalitions Relevant	5.42 (1.78)	5.22 (1.35)	0.20 (2.67)	58	0.59	.56	.08
3b: Coalitions Irrelevant	6.18 (1.80)	4.80 (1.20)	1.38 (2.71)	79	4.56	10^{-5}	.46
**Color conditions - Female targets**							
1b: No coalitions	5.70 (1.73)	3.78 (1.50)	1.93 (2.44)	75	6.78	10^{-8}	.62
5b: Coalitions Relevant	5.42 (2.13)	4.85 (1.56)	0.57 (3.03)	60	1.46	.15	.18
5b: Coalitions Irrelevant	5.77 (1.84)	5.19 (1.37)	0.58 (2.78)	84	1.93	.057	.21

Table S-2. Coalitional categorization when crossed with Race (Studies 3 & 5) or Sex (Studies 4 & 6) and shirt color categorization when crossed with Race (Study 1) or Sex (Study 2)

	Errors: mean (standard deviation)						
	**same-coalition**	**different-coalition**	**difference scores**	***df***	***t***	***p***	***r***
**Gray conditions**							
3a: Coalitions Relevant ♂	6.03 (2.01)	4.85 (1.49)	1.18 (3.08)	71	3.25	.002	.36
3a: Coalitions Irrelevant ♂	5.50 (1.99)	5.35 (1.50)	0.15 (3.13)	74	0.43	.67	.05
3b: Coalitions Relevant ♀	5.82 (1.91)	4.82 (1.86)	1.00 (3.38)	58	2.27	.027	.29
3b: Coalitions Irrelevant ♀	5.71 (1.76)	5.27 (1.31)	0.44 (2.75)	79	1.44	.16	.16
4: Coalitions Relevant	5.85 (2.04)	4.59 (1.85)	1.26 (3.46)	66	2.98	.004	.34
4: Coalitions Irrelevant	5.31 (2.18)	5.16 (1.41)	0.15 (3.08)	70	0.42	.67	.05
**Color conditions**							
5a: Coalitions Relevant ♂	7.53 (2.99)	3.23 (2.67)	4.31 (5.35)	72	6.88	10^{-8}	.63
5a: Coalitions Irrelevant ♂	6.15 (1.92)	4.62 (1.67)	1.53 (3.24)	70	3.97	.0002	.43
5b: Coalitions Relevant ♀	7.45 (3.10)	2.73 (2.43)	4.72 (5.04)	60	7.32	10^{-9}	.69
5b: Coalitions Irrelevant ♀	6.21 (2.07)	4.75 (1.68)	1.47 (3.37)	84	4.00	.0001	.40
6: Coalitions Relevant	8.02 (3.60)	2.20 (2.58)	5.83 (5.71)	68	8.47	10^{-8}	.72
6: Coalitions Irrelevant	6.19 (1.96)	4.85 (1.64)	1.34 (3.14)	68	3.55	.0007	.40
**Shirt color categorization without coalitions**							
1a: No coalitions ♂ *	5.59 (1.99)	4.88 (1.24)	.71 (2.59)	68	2.28	.026	.27
1b: No coalitions ♀ *	4.88 (1.67)	4.61 (1.37)	.28 (2.24)	75	1.08	.29	.12
2: No coalitions	4.99 (2.02)	4.93 (1.39)	0.06 (2.57)	67	0.19	.85	.02

♂, ♀ denotes sex of target stimuli. *These are the only analyses in which results differed by sex of participant. Each sex categorized the shirt color of opposite sex targets, but not same-sex targets (see S-3 and main text).

Table S-3. Shirt color categorization without coalitions; male targets (Study 1a) and female targets (Study 1b)

	difference scores: mean (standard deviation)	*df*	*t*	*p*	*r*
**1a: Male targets, No coalitions**					
All participants	0.71 (2.59)	68	2.28	.026	.27
Male participants only	0.047 (2.06)	31	0.13	.90	.02
Female participants only	1.28 (2.88)	36	2.71	.01	.41
**1b: Female targets, No coalitions**					
All participants	0.28 (2.24)	75	1.08	.29	.12
Male participants only	0.80 (2.34)	39	2.16	.037	.33
Female participants only	-.31 (1.99)	36	-0.92	.36	-.15

Table S-4. Gender categorization with Coalitions Absent vs. Present (Study 2 vs. Study 4, 6)

	Errors: mean (standard deviation)						
	same-sex	different-sex	difference scores	*df*	*t*	*p*	*r*
**Gray conditions**							
2: No coalitions	6.54 (1.97)	3.08 (1.63)	3.46 (2.82)	67	10.14	10^{-14}	.78
4: Coalitions Relevant	6.74 (1.92)	3.70 (1.30)	3.04 (2.73)	66	9.10	10^{-12}	.75
4: Coalitions Irrelevant	6.68 (1.88)	3.79 (1.59)	2.89 (2.86)	70	8.52	10^{-11}	.71
**Color conditions**							
2: No coalitions	6.43 (1.67)	3.50 (1.64)	2.93 (2.33)	67	10.34	10^{-14}	.78
6: Coalitions Relevant	6.65 (2.37)	3.57 (1.24)	3.07 (2.77)	68	9.22	10^{-13}	.75
6: Coalitions Irrelevant	7.17 (1.98)	3.93 (1.32)	3.24 (2.87)	68	9.37	10^{-13}	.75

Sentences used as stimuli

Coalitions present conditions (Studies 3-6)

Introductory information

You are about to view a conversation between members of two charitable organizations. One group is with Habitat for Humanity, the other with Partners in Health. Habitat for Humanity is an organization dedicated to creating affordable housing for people who are either homeless or living in dilapidated, substandard housing. The Habitat team members are volunteers. They go where housing is needed and work together building homes. Partners in Health is an organization dedicated to eradicating hunger and malnutrition in needy households and communities. Malnourishment and severe vitamin deficiencies - the natural consequences of hunger - lead to various health problems including birth defects and stunted growth. Partners in Health team members - also volunteers - travel to poor rural communities where they provide food and nutritional supplements, such as multivitamins and protein powder, along with agricultural advice and assistance.

Each group knew they'd be sharing part of a bus ride with members of the other organization. Although both have done extensive work in the same areas of the country, this is the first time they are meeting and learning about each other. Their conversation on the bus was recorded, and you will be seeing a portion of it.

We are interested in the impressions that these volunteers make on you as they have their discussion. You will see their photographs paired with what they said.

The photos you will be seeing will advance automatically, without your having to press anything. After you have completed viewing the photos and statements, you will receive further

instructions on the computer screen. If you have any questions, you may ask them now. Otherwise, click on "OK" to begin.

Statements presented with target photos

Full statements were presented during the encoding phase. During recall, the portions that are underlined were presented in the coalitions relevant conditions; the portions that are italicized were presented in the *coalitions irrelevant* conditions. Participants were in one condition or the other. (No underlining or italics appeared in any of the stimuli presented to participants.)

1. *Hey, it's good to meet you! We heard you'd be on the same bus as us and we were looking forward to meeting you. We just finished up a long stay in the backcountry of Kanawa County* building houses for poor folks. How about you?
2. We were actually down that way a few months ago. The farmers were having a lot of trouble with their crops. We took vitamins, some canned goods, seeds, and helped with planting. *The last few weeks, though, we were down at the other end of the state. They're at the foot of the mountains down there and were hit with a ton of rain.*
3. Yeah, we built a few barns down there a few months ago. *There was a ton of flooding. The river rose twelve feet over its banks and carried a few barns and animals away. We almost lost one of our trucks. Did you guys see all the water damage?*

4. *Well, we saw what was left over from it. It was crazy. We had to move thousands of pounds of driftwood and dirt before doing anything.* *It sucked.* It really doesn't help to have your fields buried and your crops washed away when your kids are already hungry.

5. I'll bet. The houses were a wreck too. Holes in walls, collapsing roofs, everything. *We had to do some massive door-to-door recruitment: we tried to get every able-bodied person in the area to help, even people miles away, most without phones. Everyone was really cooperative. It's cool that you guys got down there. So what do you usually do when you get to a new place?*

6. First we give out vitamins and food and then figure out priorities. Then we check and fix the water supply, we do soil and groundwater analysis. We also have some pretty cool new computer-based technology for finding nutritional problems and figuring out which crops would be best for both the soil and the people. *So pretty much we start with the simple stuff first and then hit the longer-term projects as hard as we can.*

7. *So how do you guys pick where you're going to go? Our projects get planned by our office. But from that point on we're pretty much left on our own to coordinate equipment and materials.* Then we build or repair a house or a couple houses within a few weeks or even days.

8. *We're as much scouts as we are the cavalry. Usually the police and government agencies in the area have a pretty good idea about the condition of most settlements, even in really rural areas. But in the course of being out in there we sometimes come across tiny little places completely off anyone's map. It's kind of creepy for us sometimes.* These are usually the places where people are literally starving, especially older people.

9. *Wow. We don't really come across anything like that. It's crazy to think there's actually unexplored civilization in this country. Our problem is getting done what we need to do once we get somewhere. We all get pretty frustrated with each other, but we really depend on and trust each other, we have to.* Building or repairing a whole house is tough and dangerous, especially with a deadline. Jason [Kylie] here broke his [her] thumb handling some old planks, and we've almost all fallen through old floors or gotten smacked on the head.
10. Yeah, we have to make sure no one runs off with our food, especially the black bears! It's also dangerous for kids to get overdoses of vitamins, so we have to watch them. *Sometimes we also have trouble with the weather. We have a lot of equipment that can't get wet. We have tents and everything, but we usually have to use big temporary shelters for us and our equipment.*
11. We've all been through carpentry school, or have been subcontractors, or have worked construction. *You guys have any special training? You must. We have a skill-based requirements and a long training program. We also ask for a pretty big time commitment.*
12. Some of us are nutritionists, some trained in agriculture. Some of us are specifically trained on the computer programs and the instruments for measuring soil quality and nutrition levels. *We've all had to have some minimal level of training. We work together on the big general projects, and then based on our backgrounds and experience we also each specialize.*
13. *Sounds impressive. Your work sounds a lot like ours, using your muscles as much as your brains. We've been working together for over a year. Like a well oiled machine by now.*

Built a house in less than a day once. We did everything: the electrical, the plumbing, the siding, and the roof. We were throwing boards around all day.

14. *That's incredible! You all got that done all in one day? We're lucky to get everything set up in a day. Though I suppose what we're trying to do is a bit different. We have to do a lot of teaching, a lot of talking to people.* Find out what they've been eating, where they've been planting, what health problems they've been having. It makes it hard because you have to find out so much about a place before you can do any long-term help.
15. *Well we do have to do a lot of planning. We have very specific plans before we go anywhere. That planning is long and tedious, and can take weeks if not months. I promise it's not easy.* We have to go over what we're going to build, make blueprints, order supplies, and even worry about things like road conditions.
16. *I'll bet. We've gotten stuck on lots of backcountry roads. We once almost flipped a car down a ravine.* It was full of our computer equipment, vitamins, and us. We were lucky.
17. We usually drive big trucks full of really heavy lumber, roofing tiles, and tools. *We were constantly getting stuck until we got equipment for getting unstuck. You guys should really look into getting some winches.*
18. *That's a good idea. So who pays for all that stuff? Raising money is something we're always doing.   It's tough because the people we help aren't the people who fund us.* The supplements and food add up pretty quickly. We sometimes get local agencies to contribute by explaining that we're saving lives and money in the long run.
19. *That's true. I'm sure our work pays for itself. We're changing the daily lives of these people, which can be pretty bad in a lot of ways.* No indoor plumbing or anything. To answer your

question, we have lots of our own tools and we can usually get a cheap mortgage for the family we're helping if everything's not covered by donations.

20. *So it sounds like you do a lot of your own fundraising, too. We also save a lot of money by recruiting and teaching the local people. But of course we also have to handle a lot of things ourselves.* They identify who's pregnant, who's hungry, whose crop failed, and do a lot of the distribution. But it takes training to spot nutritional deficiencies. And to know the combinations of vitamins that pregnant women, nursing mothers, and children need.
21. Yeah, each homeowner is supposed to put some sweat equity in. Build part of it themselves. Even though they don't always have a lot of building experience *they're really happy to help. It's a lot of fun. It's great to see such a change in a place. There's such a difference between when you show up and when you leave.*
22. Tell me about it. People can barely buy enough food for their kids. *They get really desperate. Some of the families we visited had sold just about everything they owned.*
23. *I'm sure the kids loved having you guys around. They always find it's such a treat to meet new people.* Sometimes we help them build little birdhouses and projects like that.
24. Yeah, sometimes we make little gardens. The kids take so much pride in the plots we make for them. *The kids, and for the most part, all the people that we come across are really hard working, friendly, and thankful. It makes all our hard work and long days worthwhile.*

Coalitions absent conditions (Studies 1 and 2)

The content of the statements is similar to that in the coalition conditions; some phrases are identical. But the context of a random government survey in the introductory information makes it clear that these people are not members of two coalitions.

Introductory information

Several years ago, the U.S. Government wanted to establish a truly random sample of all United States citizens for survey purposes. A lottery system with social security numbers was used. Out of all the citizens in the United States, a small random sample was drawn. These individuals were contacted by local government agencies. To ensure full participation, the government offered a seven year break from all taxes. Participation rates reached 99%. Part of the survey involved collecting a photograph of each participant, a recorded interview, and the completion of several surveys.

You are about to see a sample of this random sample of people. Along with their photograph, you will see a portion of what they said in their interview. The people are not talking to one another; each statement is an independent sample from each person's pre-recorded interview. To ensure confidentiality of their statements, the government has only released "decontextualized" portions of their statements, so that no personal information is made public. It is these "decontextualized" statements that you will be seeing.

We are interested in your impressions of these individuals as they make their statements. You will see their photographs paired with what they said.

The photos you will be seeing will advance automatically, without your having to press anything. After you have completed viewing the photos and statements, you will receive further instructions on the computer screen. If you have any questions, you may ask them now. Otherwise, click on “OK” to begin.

Statements presented with target photos

1. Then she said “Hey, it’s good to meet you! I heard you’d be here and we were looking forward to meeting up with you.” They had just finished up a long stay there. As I remember, they were keeping pretty busy while they were there.
2. I realized it a few months ago. The Smiths were having a lot of trouble with their car. They brought groceries, some chairs and tables, and helped with our stuff. The last few weeks, though, they had to spend some of their time other places. They were staying somewhere pretty far away. Somewhere I think that’s recently been hit with a ton of snow.
3. They started showing up a few months ago. There was a lot of commotion then. But they’ve totally stopping showing up now. They got carried away with the whole thing. We figured it wouldn’t last. Some of us were happy they left, but a couple people seemed really upset.
4. Well, I saw what was left over from it. It was crazy. They had to move thousands of pounds of rubble and debris before doing anything. I was young but I remember that it sucked. It really doesn’t help to have your neighborhood trashed when you just moved.
5. I know the kitchens had to be replaced, too. Cracked walls, sagging roof, everything. They had to do some massive wall-to-wall renovation. They tried to hire every able-bodied person in the area qualified enough to work there, even some people who they ended up firing.

Everyone was really friendly, though. It's cool to see it now it's up and running smoothly. It's a rocking place.

6. When they lost the medication, he was pretty upset. Said something about checking priorities and keeping track of things a lot more carefully. They wanted to redo the entire system, so that kind of thing wouldn't happen again. They bought some new computers for inventory tracking. He hasn't had any problems since. They seemed to care and actually wanted to fix problems. Things do seem to be better now. You can imagine, he's totally relieved.
7. I don't know how you pick what you're going to do. You know plans don't always fit with what happens. But, more or less, it's all worked out, even on their own. Been pretty fortunate. There've been ups and downs, but it seems to me to have worked out just fine so far.
8. She had mentioned something about scouts and the cavalry. Pretty strange stuff. Usually the police and government agencies in the area have a pretty good idea about that kind of thing. But in the course of being out there you sometimes come across places completely off anyone's map. It's kind of creepy sometimes. These are the kinds of places where people are literally starving, especially older people. Totally crazy to think about, isn't it?
9. We were all frustrated with each other, but we really depend on and trust each other, we have to. Making sure everyone is staying safe is tough and dangerous, especially with everything going on. We've all almost gotten hurt, but you watch your back, and everyone else's, and everyone does the same for you. You just do it, and try not to think about it too much. You know, there are always people who get unlucky, or make bad decisions, or don't pay attention, but you try not to think about that.

10. It was cool, we saw lots of animals. The kids liked seeing them, especially the black bears. It was kind of dangerous for kids to get too close, so you have to watch them. The weather got pretty bad. They have a lot of stuff that can't get wet. They had tents and everything, they even made big temporary shelters. It was pretty impressive. I'd definitely recommend it to anyone.
11. They've all been through high school already, or have been out working, or started college. The place has a lot of skill-based requirements and a long training program. They also ask for a pretty big time commitment. They've been really busy.
12. Some have worked hands on, others have more formal training, or worked for companies. A few were trained on the computer programs and the instruments for measuring and diagnosis and stuff. They've all had to have some minimal level of training. They work together on the big general parts, and then based on background and experience, they also specialize.
13. I thought it sounded a lot like of fun, using your muscles and your brains. I got interested in it. I've been doing it for over a year now. Like a well oiled machine by now. I can do things I couldn't even a couple months ago. It's been a great time. I'm sure I'm going to keep it up.
14. He said "It's incredible! I've never been experienced anything like it." Though I suppose he had to wait a long time for it. He does a lot of teaching, a lot of talking to people. Works hard to get different points of view on it. I'm sure it was tough to do because you have to find out so much before you can go ahead, but I'm sure he's happy now he went through with the whole thing. I'm not sure how he did it.
15. We do have to do a lot of planning. We usually have specific plans before we start. It can be long and tedious, and can take weeks if not months. I promise it's not easy. We have to go

over where we're going to be, make some kind of plan, buy supplies, and even worry about things like roads.

16. She'd gotten stuck on some backcountry road. Don't remember exactly where. And once almost flipped the car down a ravine. It was dark, raining, and hard to see. She was really lucky.
17. All those big trucks full of really heavy stuff like lumber and roofing tiles make a lot of noise. It really bothers a lot of people and now they're trying to do something about it.
18. Looking at it that way, it seemed like a good idea. But I wasn't sure who paid for it all. Raising money was never one of their strengths. I think it was tough because the people who got helped weren't the kind of people who had money to donate back to them. All that stuff adds up fast.
19. I'm sure her two jobs paid for a lot. It did affect our day to day lives in a lot of ways, some good, some bad. No long weekends at home or anything. To answer your question, we have lots of relatives we stay in touch with and we can usually find someone in the family to help us out. It's okay.
20. There were tons of people there. We also save a lot of money by using second hand and sharing stuff when its needed. But of course we also have to handle a lot of things ourselves. Some of us referee on a rotating basis, when we're not playing. But it takes some skills to know how to make calls. Plus, not everyone can handle keeping track of the timing and the score.
21. They really put some sweat and blood into it. Took care of it all themselves. Even though they don't always have a lot of help from my siblings, they're really happy to help. It's been

a big burden off. It's great to see such a change in a place. There's such a difference between a couple years ago and now.

22. I was trying and trying to contact them, but they went out of business. They seemed really desperate. I heard some of the families sold just about everything they owned.
23. I'm not sure what kids think about being around that kind of thing. They always seem to do okay. Sometimes those teachers help them build little birdhouses and projects like that.
24. Sometimes I stay pretty late. The streets are safe at night, and for the most part, all the people I've come across are really good people, friendly and decent. It's made the move and all the other changes worthwhile. I wouldn't go back and change it, even if I could.
